# Inferring Gene Regulatory Network Architecture Underlying Complex Traits: An Integrative Analysis of Mutant Lifespan and Gene Expression Profiles Identifies Master Regulators and Key Functional Modules for Yeast Aging

**DOI:** 10.1111/acel.70511

**Published:** 2026-04-25

**Authors:** Meng Ma, Juan Long, Yuting Chen, Yanqiu Shao, Man Zhu, Jiashun Zheng, Lu Yang, Yin Xiao, Matt Kaeberlein, Brian K. Kennedy, Yi Zheng, Hao Li, Jing Yang

**Affiliations:** ^1^ Department of Health Management & Institute of Health Management, Sichuan Provincial People's Hospital, School of Medicine University of Electronic Science and Technology of China Chengdu China; ^2^ Laboratory of Aging Research, School of Medicine University of Electronic Science and Technology of China Chengdu China; ^3^ Department of Biochemistry and Biophysics University of California San Francisco San Francisco California USA; ^4^ School of Pharmaceutical Sciences Tsinghua University Beijing China; ^5^ Optispan, Inc. Seattle Washington USA; ^6^ Healthy Longevity Translational Research Programme, Yong Loo Lin School of Medicine National University of Singapore Singapore Singapore; ^7^ Departments of Biochemistry and Physiology, Yong Loo Lin School of Medicine National University of Singapore Singapore Singapore; ^8^ Centre for Healthy Longevity National University Health System Singapore

**Keywords:** complex traits, core genes, LASSO regression model, master regulators, MSB3, peripheral genes, replicative lifespan

## Abstract

Complex phenotypes, including aging, are influenced by a connected gene regulatory network with many interacting nodes. It has been proposed that some genes, termed “core genes,” directly contribute to a trait, whereas “peripheral genes” influence the trait indirectly through network interactions. Yet demonstrating such a layered architecture and assigning genes to layers remains challenging. Using yeast aging, we developed an approach to infer network architecture underlying complex traits. Through analysis of lifespans and gene expression profiles of yeast deletion strains, we identified master regulators (MRs) whose expression change accounts for lifespan changes across mutants. Experimental tests validated 7 out of 9 MRs predicted to extend lifespan with reduced expression, and 2 out of 2 MRs predicted to extend lifespan with increased expression. We define peripheral genes as those whose effect on lifespan can be accounted for by MRs. We explored downstream mechanisms for lifespan extension by analyzing expression profiles of lifespan‐extending MR mutants. We identified a set of altered functional modules—groups of core genes that work together in biological functions, such as stress response, autophagy, proteostasis, and ribosome biogenesis. These modules were validated by single‐cell studies using one MR as an example. Our study reveals a network architecture where peripheral genes link to MRs, which connect to functional modules of core genes to influence lifespan, generalizing the previously proposed peripheral/core gene architecture. Our approach may be applied to analyzing complex human traits by integrating genetic perturbation vs. phenotype and expression data, such as those from GWAS and eQTL studies.

## Graphical abstract

We developed a LASSO‐based approach to uncover a three‐layered “fan‐in fan‐out” network architecture for complex traits like yeast aging, showing that peripheral genes influence lifespan indirectly through master regulators, which converge on core functional modules (e.g., stress response, autophagy). This framework links genetic perturbations to phenotypes and expression data, offering a strategy for dissecting complex human traits.

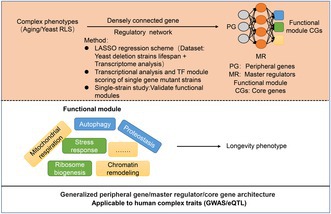

## 1 Introduction

Complex phenotypes are generally influenced by a densely connected gene regulatory network with a large number of mutually interacting nodes. As a result, perturbation of many nodes in such a network can affect the phenotype either directly or indirectly mediated by other nodes through the interaction network. Because the structure of such regulatory networks in general is poorly characterized, genetic analysis typically identifies a large number of seemingly unrelated genes. This significantly limits the power of genetic analysis in delineating the molecular mechanisms underlying complex traits.

One class of complex phenotypes is human traits, such as human height. Systematic genetic analyses through genome‐wide association studies (GWAS) with large cohort sizes revealed a large number of loci that contribute to inheritability; these loci are distributed throughout the genome and implicate many genes with a diverse range of functions. Similar observations were made based on GWASs of other complex human traits, such as schizophrenia (Weiner et al. 2023). To explain these observations, an omni‐genic model of complex traits has been proposed (Boyle et al. 2017; Liu et al. 2019). This model posits that there is a set of core genes mechanistically linked to the phenotype, and many genes associated with the phenotype indirectly by interacting with the set of core genes through a densely connected gene regulatory network (called peripheral genes). This proposal offers a conceptual framework to think about the genetic mapping of complex traits and highlights the importance of classifying the network nodes with different properties. However, it is quite challenging to prove the existence of different classes of nodes and find practical ways to identify them using human traits, as our knowledge of the network underlying these traits is scarce.

**FIGURE 1:**
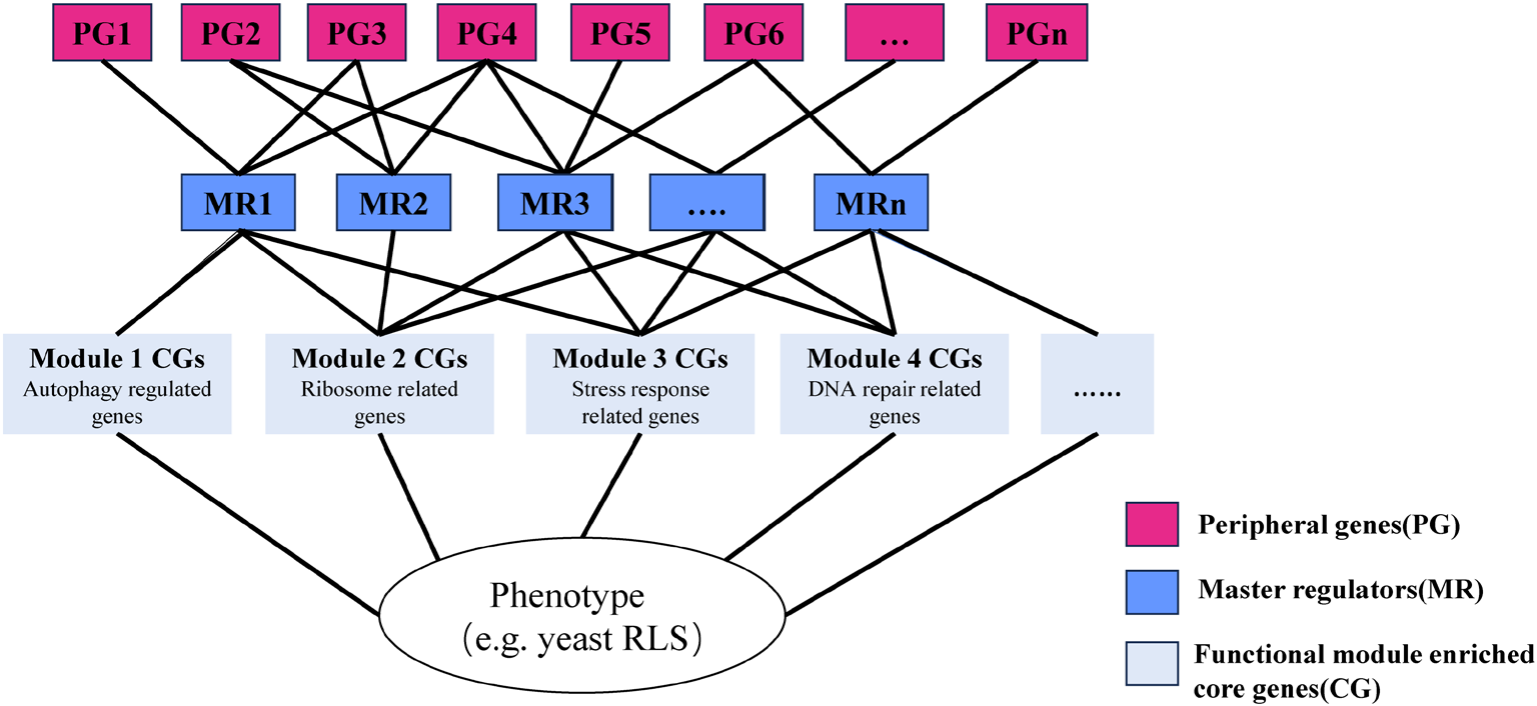
The schematic of the basic concept of the three‐layer omnigenic model that underpins complex phenotypes.

Here we generalize the basic concept of the omni‐genic model and apply it to a different complex phenotype: the replicative lifespan of yeast. Yeast replicative lifespan is arguably one of the most complex phenotypes. Previous systematic genetic analysis identified over ~200 non‐essential genes whose deletion extends lifespan (McCormick et al. 2015); most of these genes are seemingly unrelated orphan genes. The list of genes that influence lifespan is likely to grow as the analysis extends to a large number of cells to reveal genes with a smaller effect size. We posit that most of these genes with lifespan‐extending phenotypes exert their effect indirectly through the network, i.e., they are peripheral genes. Generalizing the concept of omni‐genic model, we hypothesize that besides the “core genes” that are mechanistically linked to the phenotype (e.g., proteasome genes that help maintain proteostasis or DNA repair genes that maintain genome integrity) and “peripheral genes”, there is an additional class of nodes we called “master regulators”, that bridge peripheral and core genes, and that can be grouped into different functional modules (Figure 1). The hypothesis is based on previous observations that aging is associated with the dysfunction of multiple cellular processes, and that to extend the lifespan of the cells, multiple functions need to be improved in concert (López‐Otín et al. 2023). Thus, the perturbation of a single core gene may not be able to extend lifespan. Instead, the perturbation of a “master regulator” that controls multiple “core” genes is needed. This concept of an additional layer of master regulators could be generalized to other complex phenotypes using the same rationale.

We chose yeast replicative aging as the complex trait to test the above hypothesis because there is a large amount of lifespan and omics data under various genetic perturbations that can be used to reconstruct gene regulatory networks, and many genetic tools are readily available for follow‐up studies. In addition, yeast replicative aging has been a canonical model for aging studies and has contributed significantly to the important pathways that regulate lifespan across species. Thus, analysis of the lifespan phenotype from this network perspective may shed additional light on the mechanisms of cellular aging.

With the above model in mind, we developed a mathematical approach to identify the master regulators by combining the systematic lifespan data with gene expression profile data for a number of gene deletion mutants. Assuming that the mutant lifespan phenotype is mediated through gene expression changes, we identify a small set of master regulators whose expression changes contribute significantly to the lifespan of multiple deletion mutants. For these master regulators, we predicted the direction of perturbation (increase or decrease the expression) that can extend lifespan, and our subsequent experimental tests show that our predictions are highly accurate: 7 out of 9 predicted negative lifespan regulators (reduced expression is predicted to extend lifespan) and 2 out of 2 predicted positive regulators significantly extended lifespan. In contrast, only 1 out of 20 genes picked randomly from the genome has a lifespan phenotype (McCormick et al. 2015). Most of the identified master regulators are not covered by the original lifespan data (the non‐essential gene deletion collection) used to perform the model training and thus are novel predictions.

To examine the structure of the network, we connected the upstream peripheral genes with our validated master regulators based on our computational analysis and connected the master regulators to their downstream core genes by generating additional gene expression data with the master regulator perturbed. Consistent with the model we proposed, we found that many of the peripheral genes are linked to one or more master regulators, and different master regulators are linked to sets of core genes with strong overlap, covering a broad spectrum of mechanisms known to be important for aging. Our study provides support for the existence of the three‐layered structure of the gene regulatory networks underlying complex traits and suggests practical approaches to identify the master regulators. Although we used yeast replicative aging as a model, we believe the general concept and similar mathematical approaches can be applied to complex human traits, as more genetic variations vs. phenotypes (from GWAS) and gene expression data (from eQTL) become available.

## 2 Results

### 2.1 Construction of a Mathematical Model to Identify the Master Regulators That Contribute to Yeast Longevity

**FIGURE 2:**
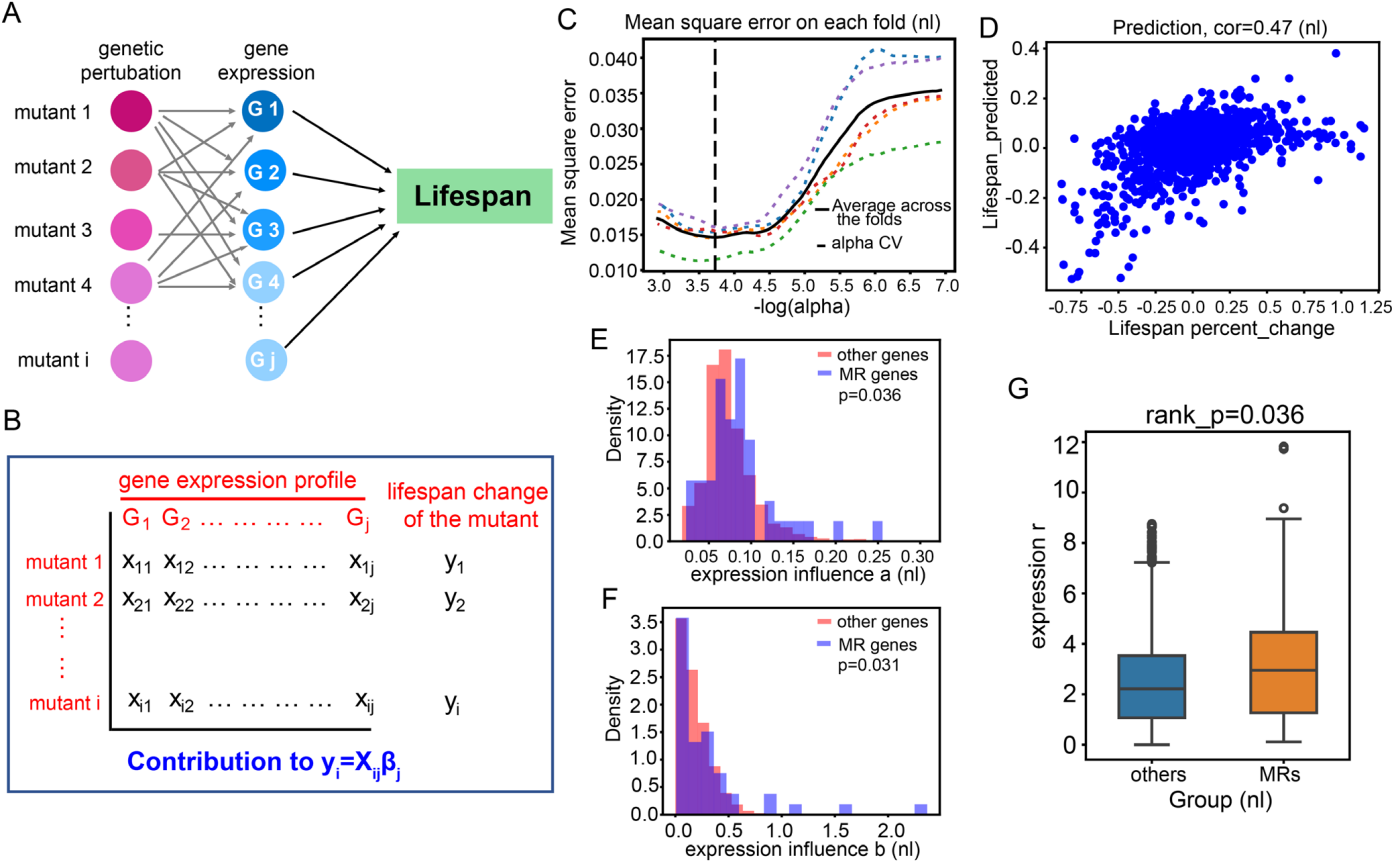
Mathematical model construction and identification of the master regulators that are involved in yeast replicative lifespan. (A) Proposed model of peripheral genes influencing RLS via regulating the expression of MRs. (B) mathematical framework for the prediction of MRs. (C) Determination of penalty parameter α via cross‐validation (non‐linear model). (D) Evaluation of the model prediction efficiency by comparing the observed and predicted RLS changes in mutant strains (non‐linear model). (E) Average magnitude of the expression change: MR genes vs. other genes across long‐lived mutants (non‐linear model). (F) Directional consistency of expression changes: MR genes vs. other genes across long‐lived mutants (non‐linear model). For E&F, we used the two‐sample Kolmogorov–Smirnov (KS) test to quantify the distributional differences. (G) Ratio of directional consistency to average magnitude of expression change for MR genes vs. other genes (non‐linear model).

With the hypothesis that peripheral genes connect to a set of MRs, which then coherently regulate a set of functional modules (groups of core genes) to influence lifespan (Figure 1), we set out to develop a mathematical model to first identify the MRs. Our basic assumption is that if a mutant changes the lifespan (relative to the wild type), it does so through the change of the genome‐wide gene expression profile, and in particular, through the change of gene expression of the MRs (Figure 2A).

In yeast, there are ~4000 non‐essential genes. The replicative lifespan of the deletion mutants of these genes has been systematically measured (McCormick et al. 2015), and a significant fraction of these mutants have been profiled at the transcriptome level (GSE42528; a dataset including 1479 mutants) (Kemmeren et al. 2014). We combined the transcriptome and lifespan data of these mutants (Figure 2B) to develop a mathematical model in which the expression change of each gene contributes additively to the lifespan change of a mutant, i.e., for a mutant_*i*, the change of lifespan *y*_*i*_ = sum_*j*_ *β*_*j*_ *x*_*ij*_, where *x*_*ij*_ is the log fold change of gene *j* in mutant *i*, and *β*_*j*_ is the strength of the contribution of gene *j* to the lifespan. We then infer *β*_*j*_ by fitting the lifespan data using the gene expression data. Since the number of data points (number of mutants with both lifespan and transcriptome data, which is 1405) is significantly smaller than the total number of possible parameters (number of *β*_*j*_ which is the number of genes in the genome ~6000), we used LASSO (Least Absolute Shrinkage and Selection Operator) regression to prevent overfitting and select the relevant parameters. We expect that MRs will be selected by the model and have significantly larger contributions to the lifespan.

Since the lifespan data for different mutants were measured from different numbers of mother cells, the reliability varies significantly across mutants. We thus developed a weighted LASSO regression scheme with the cost function described by the following formula:

$$\text{Cost}=\sum_{i=1}^{N}w_{i}{\left(y_{i}-\left(\beta_{0}+\sum_{j=1}^{p}\beta_{j}x_{\mathrm{ij}}\right)\right)}^{2}+\alpha \sum_{j=1}^{p}\left(︱\beta_{j}︱\right)$$

where $w_{i}$ is the weight for the mutant strain *i* (calculated from the number of cells used in the lifespan measurement, described below), *y*_*i*_ is the percent change of lifespan of the strain *i* relative to the wild type strain, $\beta_{j}$ is the slope of the regression quantifying the contribution of gene *j* to the lifespan, $x_{\mathrm{ij}}$ is the gene expression (log_2_ foldchange) of the gene *j* in mutant strain *i*, and alpha is the penalty parameter in the LASSO regression. We choose a weighting function:

wi=nini+50

where $n_{i}$ is the number of cells measured in the lifespan experiment. This parameter ensures that strains with smaller number of cells analyzed (e.g., $n_{i}$ < 50) receive a smaller weight, thereby contributing less to the cost function and having less influence on model fitting. Conversely, for strains with large sample sizes (e.g., $n_{i}$ > 100), the weight approaches 1, reflecting the higher confidence in the reliability of their lifespan measurements—consistent with the standard practice in the field of measuring over 100 cells per strain to obtain robust lifespan data.

To evaluate the robustness of feature selection to the specific choice of the stabilizing constant, we performed a sensitivity analysis by replacing the constant 50 with 30 and 80, respectively. As shown in Figure S1, the three sets of identified master regulators (with constants 30, 50, and 80) mostly overlap, with the vast majority of genes consistently selected. These results indicate that the regression is robust with respect to the choice of weighting factors.

As we are dealing with gene expression data for deletion mutants, the log fold change of the expression of the deleted gene itself is singular (i.e., *x*_*ii*_ will be a large negative even after adding pseudo counts). We thus used two strategies to regularize the self‐contribution (so that they will not dominate over the contributions of other genes): “self0” and “nonlinear”. In the “self0” strategy, we manually set the self‐contribution to zero, and in the “nonlinear” scheme, we translate *x*_*ij*_ to *x*_*ij*__tilde using a nonlinear function that saturates when *Ix*_*ij*_ *I* is large (see Section 4), thus limiting the self‐contribution. We found that the two different strategies lead to similar results (see below).

As is standard in LASSO regression, the penalty parameter alpha is fixed through cross‐validation by selecting the alpha that minimizes the fitting error in the test sets (Figure 2C and Figure S2A).

After variable selection by LASSO, 70 and 55 genes are left in the “non‐linear” and “self0” models, respectively. Table 1 shows the overlapping genes from the two models, including 29 and 15 genes that contribute positively or negatively to the lifespan (see Table S1 for a full list of genes and their *β*_*j*_ values from the two models). The positive contributors (increased expression leads to increased lifespan) include the well‐known NAD(+) dependent histone deacetylase SIR2 (Howitz et al. 2003), and the negative contributors (decreased expression leads to increased lifespan) include four essential genes, TYS1, TOP2, ERG25, and CDC7. Among these genes, TOP2 knockdown and SUL1 deletion were reported to significantly extend lifespan in our previous studies (Zhu et al. 2025; Long et al. 2025). Using the above genes and coefficients *β*_*j*_ (separate gene sets for the two models), we calculated the predicted RLS change and compared that to the actual RLS change for different mutants (utilizing the entire dataset as the training set). Both the “non‐linear” and “self0” models predict lifespan change reasonably well (Figure 2D and Figure S2B).

**TABLE 1:** Overlap genes (MRs) of the “self0” or “non‐linear” regressions.

	Gene name	coef1 (non‐linear)	coef2 (self0)	RLS extension (%)	cell number	Essential gene	Choose for validation
Negatively contributed genes	TYS1	−0.19761211	−0.11012192	/	/	Y	Y
	SUL1	−0.13661558	−0.13646341	12.66766	25	—	Y
	NIT3	−0.11023048	−0.0834808	13.96304	20	—	Y
	TOP2	−0.07990489	−0.02396487	/	/	Y	Y
	ERG25	−0.07835613	−0.031813	/	/	Y	Y
	SSA4	−0.07830232	−0.00081554	43.13725	5	—	Y
	MSB3	−0.0733408	−0.01332473	53.50877	5	—	Y
	CHS3	−0.05444198	−0.12216669	−38.7755	5	—	Y
	YCL007C	−0.05432505	−0.00995229	−22.807	5	—	—
	YMR013W‐A	−0.05378167	−0.01161382	/	/	Y	—
	PGM1	−0.04558591	−0.01307923	−8.73016	5	—	—
	ZDS2	−0.03854758	−0.03756222	8.22838	45	—	—
	HSP26	−0.0246761	−0.00852084	27.89116	5	—	Y
	COS12	−0.01599506	−0.00456833	9.106138	265	—	—
	CDC7	−0.00156776	−0.03263791	/	/	Y	—
Positively contributed genes	YCR001W	0.000516884	0.010225692	5.263158	5	—	—
	MSN2	0.008964552	0.015242508	4.895105	5	—	—
	ARF3	0.017033847	0.001863061	35.83333	5	—	—
	RHO5	0.01744442	0.010701765	−26.5152	5	—	Y
	YOR263C	0.017837455	0.031536908	20	5	—	—
	TFC4	0.018150573	0.000513367	/	/	Y	—
	RPS26A	0.018715249	0.013732128	/	/	Y	—
	PRE5	0.020408206	0.014862617	/	/	Y	—
	KNH1	0.023518058	0.027379698	13.76147	5	—	—
	YGL024W	0.026605089	0.00847584	26.5625	10	—	—
	RPL34B	0.032361924	0.008348967	35.39948	127	—	—
	AHC2	0.041642089	0.13383192	23.48485	5	—	—
	CWC23	0.046364215	0.014268309	/	/	Y	—
	YHR140W	0.054828286	0.011190545	−18.018	5	—	—
	RPL17B	0.055039373	0.033653052	−25.773	25	—	—
	CCS1	0.056728738	0.060189574	39.80583	5	—	—
	GEX2	0.02649678	0.002500175	55.76923	5	—	—
	YJL156W‐A	0.064897844	0.112392182	/	/	Y	—
	TSA1	0.068291418	0.026587365	−46.3309	109	—	—
	LIP1	0.073814309	0.022556348	/	/	Y	—
	PTP3	0.075648633	0.004047807	−17.6056	5	—	—
	IZH1	0.082637974	0.079801597	30.57851	5	—	—
	YNL089C	0.10545626	0.0012954	21.42857	5	—	—
	SAD1	0.118923457	0.079308253	/	/	Y	—
	HTA2	0.141827991	0.141473084	10.09174	10	—	—
	CTS1	0.158457236	0.102076776	−25.3521	5	—	—
	JID1	0.166152991	0.166882153	15.95745	5	—	—
	SIR2	0.196431307	0.124014733	−46.5657	1837	—	Y
	YGR139W	0.222199858	0.135879443	/	/	Y	—

*Note:* “/” means the data is absent in previous datasets.

Thus, through the mathematical modeling and LASSO regression analysis, we were able to identify a small set of genes whose expression changes can account for the lifespan changes of a much larger number of mutants. We call this set of genes master regulators (MRs).

To get an intuitive feeling of how these MRs differ from other genes, we performed a simple statistical analysis on how the MRs vary across long‐lived mutants compared to other genes. We chose 140 long‐lived mutants with RLS extended ≥ 15% and *p* < 0.05 that have expression profiling data. For each gene *j* in the genome, we calculated two numbers: *a* = $\frac{\sum |X_{\mathrm{ij}}|}{N}$ and *b* = $\frac{|\sum X_{\mathrm{ij}}|}{\sqrt{N}}$, where *X*_*ij*_ is the log fold change of gene *j* in long‐lived mutant *i*, and *N* is the total number of long‐lived mutant (*N* = 140 in this case). While the number *a* represents the average magnitude of the expression change across the long‐lived mutants (the responsiveness), the number *b* quantifies how consistent the direction of the change is. If the sign of *X*_*ij*_ is random, we expect *b* will have a similar magnitude as *a* (distance of a random walk scales as sqrt(N)), while if the direction is more consistent, *b* will be larger, and thus the ratio *r* = *b*/*a* will be larger. We expect that MRs will show stronger responsiveness and more consistent direction of change across long‐lived mutants. Indeed, we observe that compared to general genes, the distribution of *a* for MRs shifted to a larger value, and the ratio *r* is also larger (Figure 2E–G; Figure S2E,G). We note that due to the complexity of the regulatory feedback in biological networks, consistent directionality of expression change for a MR across all long‐lived mutants connected to the MR is not guaranteed. Rather the consistent direction may just be for a subset of mutants that share similar mechanism, perhaps in a statistically significant way.

### 2.2 Experimental Validation of the Lifespan Extending Phenotypes of MR Perturbations, Including Knockdown of Essential Genes and Over‐Expression

According to our model, perturbation of the master regulators themselves would influence lifespan in the direction we predicted. Our model predicted essential genes with reduced expression and over‐expression of genes as lifespan‐extending perturbations, although we only used non‐essential gene deletion mutant data to do the inference. This is because the deletion of non‐essential genes leads to the perturbation of both essential and non‐essential genes, and in both directions. Such information was used by the model to infer MRs together with the direction of the change (the sign of *β*_*j*_) that will lead to lifespan extension.

We selected three classes of perturbations for validation: (1) over‐expression of two positive lifespan regulators, including RHO5 and the well‐known SIR2; (2) down‐regulation of six non‐essential genes predicted to be negative lifespan regulators; (3) down‐regulation of three essential genes predicted to be negative regulators. The six non‐essential genes are SUL1, MSB3, NIT3, SSA4, HSP26, and CHS3, and the three essential genes were topoisomerase II TOP2, a cytoplasmic tyrosyl‐tRNA synthetase TYS1, and a C‐4 methyl sterol oxidase ERG25. For the non‐essential genes, we analyzed lifespan in the deletion mutants. For the essential genes, we used DAmP (decreased abundance by mRNA perturbation, see Section 4) strains to do the lifespan validation.

**FIGURE 3:**
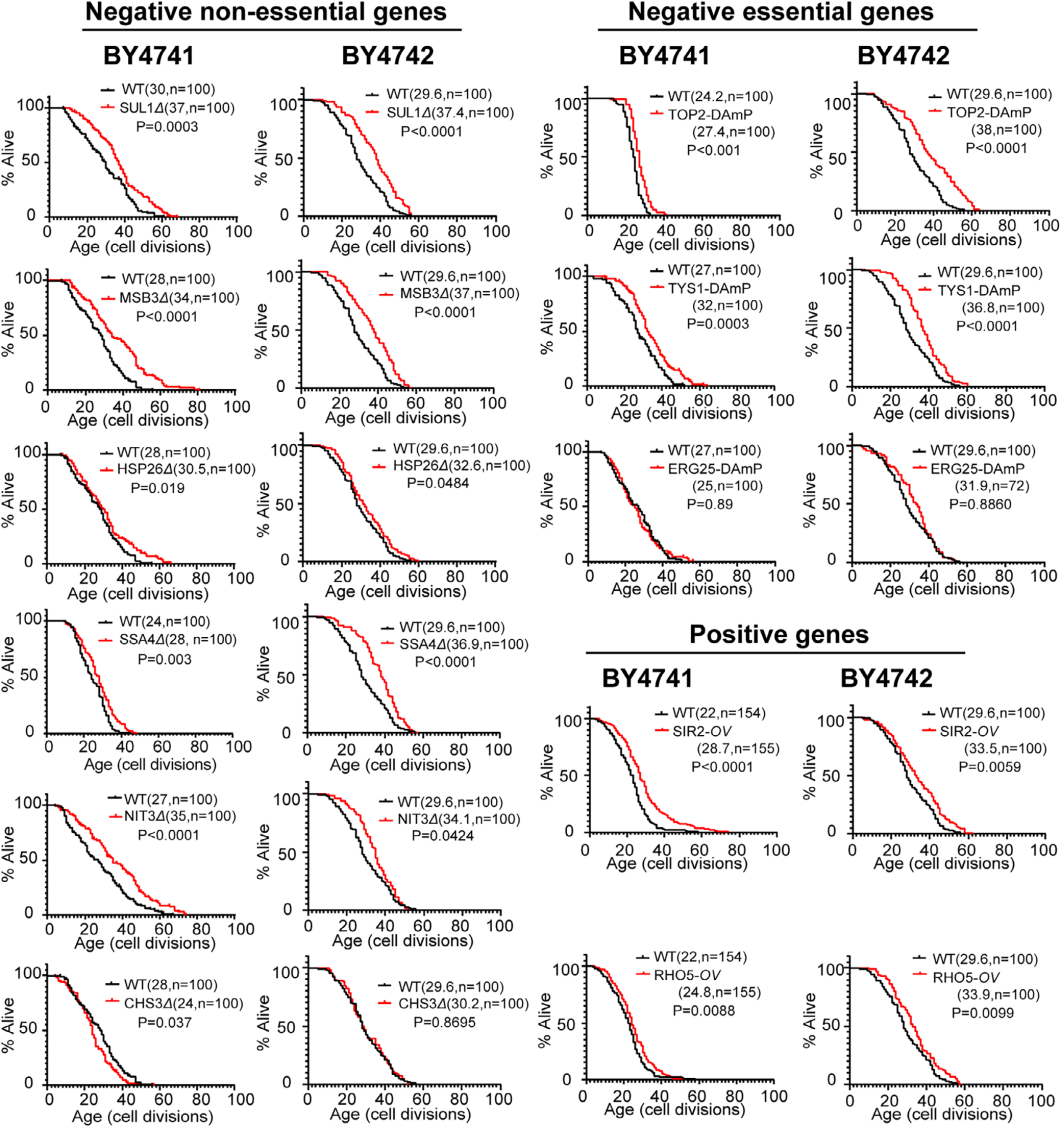
Experimental validation of the top efficient MRs in yeast RLS regulation in BY4741 and BY4742. *Negative non‐essential genes*: Non‐lethal MR genes whose decreased expression leads to increased lifespan. Negative essential genes: Lethal MR genes whose decreased expression leads to increased lifespan. *Positive genes*: MRs whose increased expression leads to increased lifespan. All statistical analyses were performed using GraphPad Prism 8.0. A *p* < 0.05 was considered statistically significant, as determined by the log‐rank test. Corresponding *p*‐values, *n* values (number of cells), and mean lifespans are indicated in each subpanel.

We find that among the non‐essential genes predicted to be negative regulators, deletion of MSB3, HSP26, SSA4, NIT3, as well as previously studied SUL1, significantly increased yeast replicative lifespan both in the *MATa* (BY4741) and *the MATα* (BY4742) strain backgrounds (Figure 3). SUL1 is a sulfate permease of the SulP anion transporter family, and our previous work found that its deletion led to decreased PKA signaling, resulting in increased stress‐protective trehalose and glycogen, increased nuclear translocation of MSN2, and elevated expression of general stress response genes, contributing to extended lifespan (Long et al. 2025). SSA4 belongs to a Stress 70 sub‐family A (SSA), which represents the major cytosolic Hsp70 family that is essential for protein quality control. HSP26 is a small heat shock protein (sHSP) with chaperone activity that forms hollow, sphere‐shaped oligomers that suppress unfolded proteins aggregation, it seems like knocking down protein quality control. NIT3 is a biofilm suppressor and a member of the nitrilase superfamily. In yeast, NIT2 and NIT3 are two proteins with similarity to the mouse and human Nit protein. NIT2 was reported to act as an excellent deaminated GSH (dGSH) amidase, thus being beneficial to cells through eliminating intermediate metabolic side products (Peracchi et al. 2017). The actual enzymatic function of NIT3 is unknown. MSB3 is a specific Rab GTPase‐activating protein (GAP) responsible for the transport of intracellular vesicles and participates in the regulation of autophagy. Deletion of MSB3 produced the strongest lifespan extension phenotype among the tested mutants (21.4% in BY4741 and 25% in BY4742) (Figure 3). We thus chose to follow the molecular phenotypes of MSB3 deletion in more detail (see the session “Mechanistic elucidation of MSB3 deletion in RLS regulation”).

For the predicted MRs that are essential genes, we performed knockdown with the DAmP technology. We found that ~50% downregulation of two essential genes, TOP2 and TYS1, significantly extended yeast lifespan in both *MATa* and *the MATα* strain backgrounds (RT‐qPCR validations of the knockdown of TOP2 and TYS1 in the DAmP strains were shown in Figure S3A). As discussed in our previous work, TOP2 reduction delayed aging and alleviated major hallmarks of aging, such as cellular senescence, DNA damage, loss of proteostasis, and deregulated nutrient sensing in yeast, *C. elegans* , and mouse (Zhu et al. 2025). We found that knockdown of the tyrosyl‐tRNA synthetase (TYS1‐DAmP) also produced a strong lifespan extension phenotype (extending yeast RLS for 18.5% and 24.3% in BY4741 (*p* = 0.0003) and BY4742 (*p* < 0.0001), respectively). These results suggest that targeting essential genes can be an effective way to increase the lifespan/healthspan and thus need more attention in future studies.

For the MRs predicted to be positive regulators, we chose 2 genes to test if overexpression extends lifespan. One is SIR2, a histone deacetylase with a well‐characterized role in regulating yeast lifespan (we used it as a positive control), and RHO5, a non‐essential small GTPase of the Rho/Rac family involved in the protein kinase C‐dependent signal transduction pathway. We found that both in BY4741 and BY4742 strain backgrounds, overexpression of SIR2 or RHO5 extends yeast RLS significantly (Figure 3). Not surprisingly, we also found that the lifespan extension with overexpression is dose‐dependent. While up‐regulating to about twofold increases lifespan, excessive expression of these genes had no or even an adverse effect on the lifespan (Figure S3B,C). twofold up‐regulation of SIR2 increased RLS by 30.5% (*p* < 0.0001) and 13.2% (*p* = 0.0059) in BY4741 and BY4742, respectively, while 2‐fold overexpression of RHO5 increased RLS by about 12.7% (*p* = 0.0088) and 14.5% (*p* = 0.0099) in BY4741 and BY4742 backgrounds, respectively.

Thus, our experiments validated the lifespan phenotype of 7 of 9 MRs predicted to be negative regulators and 2 of 2 MRs predicted to be positive regulators, indicating that perturbation of MRs predicted by our math model is much more likely to influence lifespan. In contrast, only ~3% of the non‐essential deletion strains extend lifespan (McCormick et al. 2015). In addition, functional analysis of these validated MRs suggests that they occupy special positions in the gene regulatory network. For example, Sir2 is a master regulator for genome maintenance and epigenetic regulation (Mekhail et al. 2008; Laskar et al. 2015). Top2 regulates DNA topology, transcription, and epigenetic landscape (Zhu et al. 2025). Sul1 is a trans‐receptor directly upstream of PKA signaling that regulates energy metabolism and stress response. Molecular chaperones (SSA4, HSP26) are at central hubs that interact with numerous proteins, including co‐chaperones and regulatory factors, to control protein quality and regulate stress response and cell viability (Nishikawa et al. 2001; Amorós and Estruch 2001).

### 2.3 Defining Peripheral Genes and Connecting Them to the Master Regulators

We defined MRs as those whose expression change can account for the lifespan changes across deletion mutants, identified them using the LASSO regression model, and tested the effect on lifespan of MR perturbations. Functional analysis of these genes suggests they are generally at central hubs of the gene regulatory network.

**FIGURE 4:**
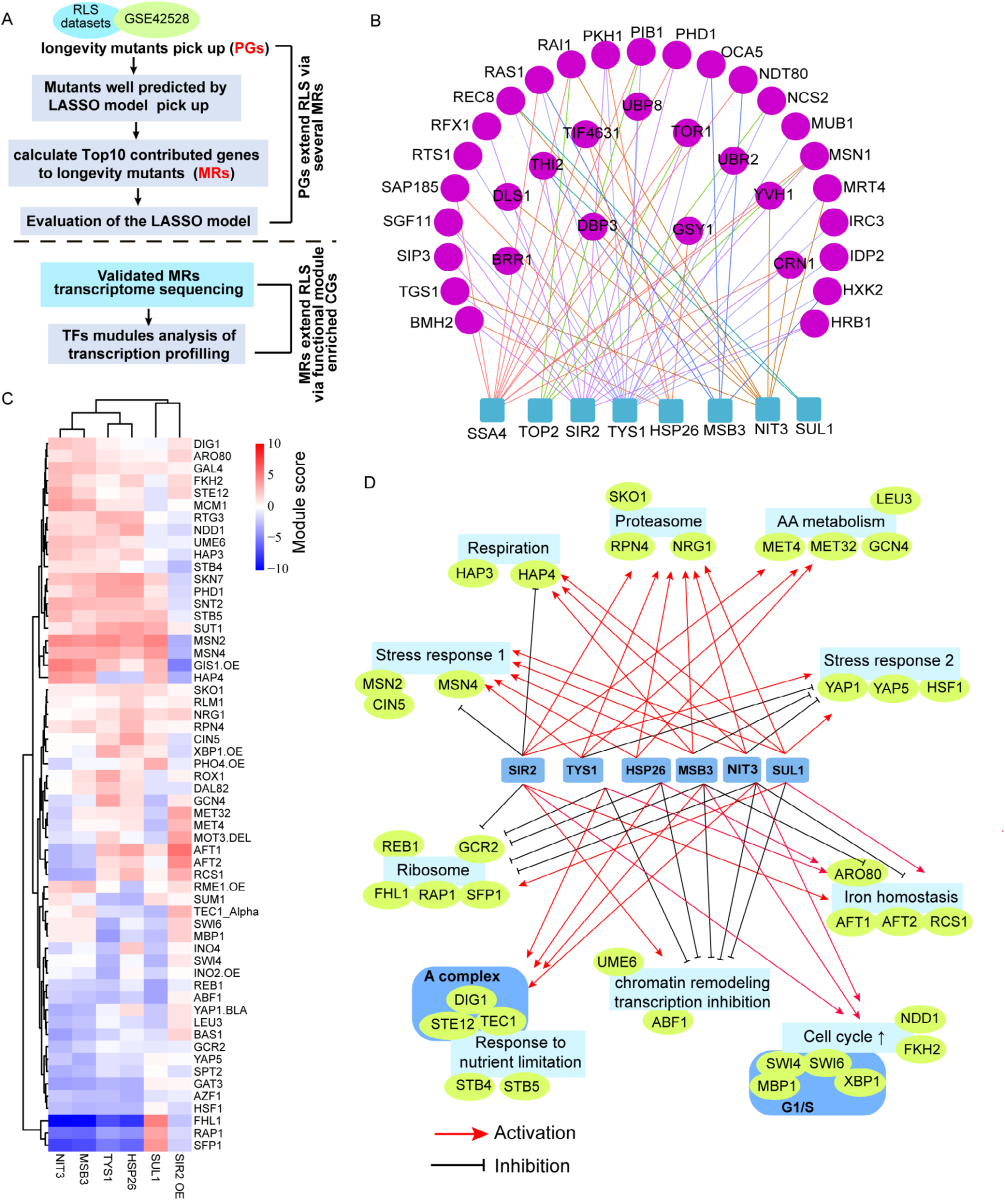
Examination of the “PGs‐MRs‐CGs” three‐layer‐network structure in yeast replication lifespan extension. (A) Work scheme of the yeast RLS “PGs‐MRs‐CGs” three‐layer network regulatory hierarchy analysis. (B) Analysis of the contribution of the experimentally validated MRs to RLS extension in long‐lived PG mutants, to evaluate the regulatory relationship between PGs and MRs. (C) TFs module score analysis of transcription profiling data of MRs (SUL1Δ, MSB3Δ, NIT3Δ, HSP26Δ, TYS1 DAmP and SIR2 OVER), to evaluate the effect of MRs on CGs. (D) Correlation network analysis between MRs and functionally enriched core longevity modules.

Here, we define peripheral genes as those whose lifespan change can be accounted for by the change of a small set of MRs. We then link these peripheral genes to the respective MRs. To do this, we investigated the contribution of predicted MRs to the extension of the long‐lived mutants using the regression model (*β*_*j*_ *X*_*ij*_) (Figure 4A,B). We first pick up longevity mutants from the lifespan and gene expression datasets, with the criteria that the gene deletion can extend RLS more than 15% with a *p* < 0.05, and the mutant has gene expression profiling data. We then select mutants whose lifespan is well predicted by our LASSO regression model (selection criterion: |L1‐L2|/(|L1| + |L2|) < 0.5; L1 = real RLS, L2 = predicted RLS). We define peripheral genes as those whose lifespan is well predicted by the model and is not a master regulator.

To connect the peripheral genes to MRs, we calculated the contribution of the *j*_th_ MR to the *i*th mutant lifespan *x*_*ij*_ *β*_*j*_, ranked the contribution, and chose the top 10 contributing MRs to connect to the peripheral gene. We found that the 34 well‐predicted longevity mutants are connected to 40 MRs (“non‐linear” model), which include most of the MRs we experimentally tested. Notably, we found that each MR is connected to many peripheral genes, while some PGs are connected to more than one MR, and some of them are connected to both positive and negative MRs (Figure S4B, Figure 4B). Interestingly, deletion of PGs like DBP3, NCS2, MRT4, RAI1, IRC3, and YVH1 can both increase the expression of positive MRs and down‐regulate negative MRs (data not shown), suggesting that the contribution of MRs to the lifespan of the peripheral genes can be coherent. Among them, YVH1, MRT4, and RAI1 showed a significant longevity phenotype. YVH1 encodes a phosphatase involved in cAMP‐mediated signaling and positively regulates pre‐autophagosomal structure formation upon rapamycin treatment (Yeasmin et al. 2015). Both YVH1 and MRT4 are involved in ribosome stalk assembly, and the latter is essential for the recruitment of translation factors (Lo et al. 2009).

The PGs and their connections to MRs presented in Figure 4 were defined by using specific thresholds for how accurate the model predicts the lifespan (|L1‐L2|/(|L1| + |L2|) < 0.5) and the degree of contribution from MR to PG (top 10 contributing MRs). To examine the sensitivity of the results with respect to the choice of thresholds, we examined different thresholds for PG assignment: 0.3 (more stringent), 0.5, 0.7 (less stringent), and different thresholds for making connections: top 5 (more stringent), top 10, top 20 (less stringent). As expected, with more stringent thresholds, we get less PGs and less connections from PGs to MRs, while with less stringent thresholds we get more PGs and more connections (Figure S4 and Tables S2–, S4). However, we found that a core set of PGs and their connections to MRs are robust with respect to the parameter choices.

We examined some of these PG to MR relationships with literature and database searches and found interesting common themes underlying them (Teixeira et al. 2023; Stark et al. 2006; Hu et al. 2018). For examples, several PGs are robustly connected to the MR Sir2, including TOR1, SGF11, PKH1, and RFX1. TORC1 signaling directly regulates SIR2 phosphorylation via CK2 kinase, and they exert opposing effects on autophagy to influence the aging process (Guedes et al. 2017; Devare et al. 2020). Since TORC1 is a major positive regulator of cell growth and SIR2 functions on genomic stability and genome maintenance, this relationship indicates a mutual inhibitory relationship between cell growth and maintenance/stress response. Consistent with this observation, our analysis indicates that TOR1 deletion strain has increased transcriptional level of SIR2 which contributes to the lifespan extension. SGF11 encodes a structure subunit of the SAGA complex which promotes transcriptional elongation. Deletion of SGF11 leads to defects in transcriptional elongation and decreased growth (Ingvarsdottir et al. 2005; Samara et al. 2010). PKH1 encodes a serine/threonine protein kinase which is an upstream activator of Sch9 (TORC related growth signaling). Deletion of PKH1 likely extends lifespan through a mechanism similar to TOR1 deletion. RFX1 is a transcriptional repressor of DNA damage response. Deletion of RFX1 likely activates DNA damage response and stress response in general and slows down growth. Thus, a common theme among these mutants is increased stress response and decreased growth. The fact that all these genes are linked to SIR2 (i.e., deletion leads to increased SIR2 expression) suggests that increased genomic stability contributes to lifespan extension across these mutants.

### 2.4 MRs Are Connected to Downstream Core Genes Organized Into Functional Modules to Influence Lifespan

Aging is a complex phenotype, and lifespan extension requires an orchestrated change of genes mechanistically linked to lifespan. According to the previous proposal, these genes are called core genes (Boyle et al. 2017; Liu et al. 2019). We hypothesize that the core genes are organized into functional modules connected to MRs (Figure 1). Mapping these functional modules is important for a mechanistic understanding of what controls lifespan. We thus analyzed the downstream transcriptional programs induced by the lifespan extending MR perturbations (Figure 4A,C,D). We performed the transcriptional profiling in the BY4741 background of the confirmed MRs, including MSB3, NIT3, HSP26, and TYS1, combined with our previous profiling data of SUL1(GSE215287). Tables S5–S8 show transcriptional profiling of MSB3Δ, NIT3Δ, HSP26Δ, TYS1 DAmP, and WT. The DEGs (different expressed genes) contain 25,22,79,66 down‐regulated genes and 45,27,64,47 up‐regulated genes in MSB3Δ, NIT3Δ, HSP26Δ, and TYS1 DAmP (FC > 1.5 or < 0.7, *p* < 0.05) deletion, respectively. We find few overlaps between DEGs identified from the above analysis, except for CGR1, part of the pre‐ribosome and large subunit precursor, which was decreased in all four mutants.

We then use a TF module score (organizing genes into functional transcription modules, sets of genes regulated by the same transcription factors) to quantify the collective expression change of genes in a TF module (see Section 4). We systematically analyze the response of different TF modules to different lifespan‐extending MR perturbations, and we added SIR2 overexpression profiling datasets here (see Section 4). We observed a consistent pattern across the lifespan‐extending MR perturbations (with a couple of exceptions, such as SUL1 deletion and SIR2 overexpression) (Figure 4C). It is clear that most of the TF modules were similarly shifted in all negative contributing MRs, but shifted in opposite directions in SIR2 overexpression, suggesting two basic mechanisms for lifespan extension (Figure 4C).

It is worth noting that these significantly altered transcription modules were enriched for several functional classes, including the proteasome, respiration, stress response, ribosome biogenesis, cell cycle regulation, and amino acid metabolism. Furthermore, each MR perturbation was found to alter the expression of several functional classes that include multiple related TFs. We thus connected the MRs to the downstream functionally organized core gene classes (Figure 4D).

Among the altered functional core classes, ribosomal genes showed strong and consistent down‐regulation. Given the important role of ribosomal genes in aging, we performed a statistical analysis to compare the change of ribosome genes vs. all the other genes in all the longevity mutants (see Section 4) and found that the expression of ribosome genes was significantly down‐regulated relative to other genes (Figure S5).

Consistent with our analysis of PG to MR connections, the TF module analysis also suggests that most of the long‐lived mutants are in a slower growth and more stress resistant state, characterized by decreased ribosomal gene expression (the FHL1, RAP1, SFP1 modules), increased stress response (MSN2/MSN4 modules), respiration (HAP3/4 modules), and proteosome expression (RPN4 module).

### 2.5 Mechanistic Elucidation of MSB3 Deletion in RLS Regulation

To further elucidate the mechanistic role of MRs in longevity regulation, we selected MSB3, a Rab GTPase‐activating protein known to play a critical role in the regulation of endocytosis and vacuole fusion processes.

**FIGURE 5:**
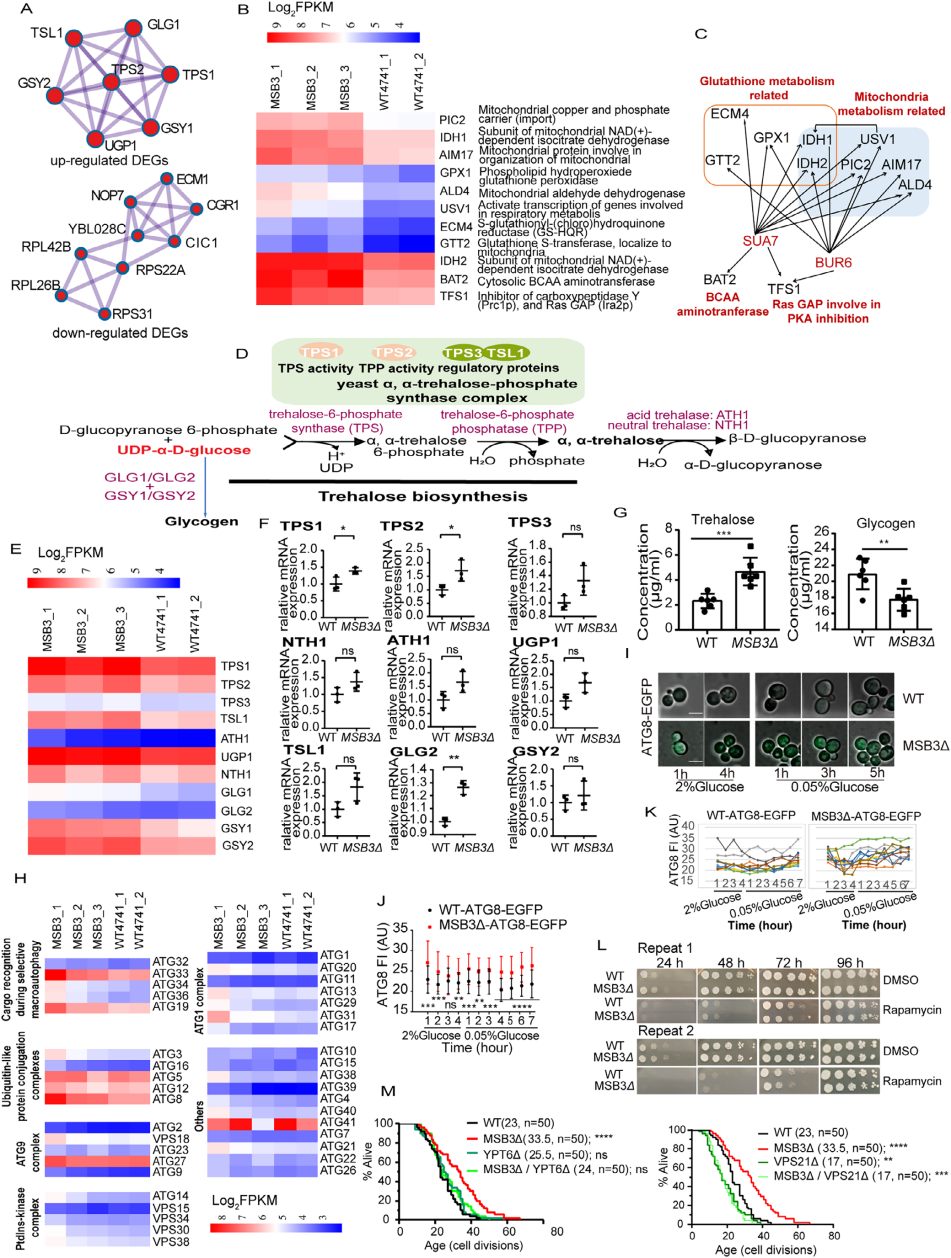
Mechanistic study of lifespan extension in a typical example of MRs MSB3. (A) PPI network analysis of enhanced trehalose/glycogen‐associated genes (above) and decreased ribosome‐associated genes (below) in MSB3Δ DEGs. (B) Heatmap of mitochondrial metabolism/antioxidant genes expression profile in MSB3Δ vs. WT. (C) Transcription regulatory network linking different expressed genes to putative TFs in MSB3Δ. (D) Schematic of trehalose/glycogen metabolic pathways in *Saccharomyces cerevisiae*. (E) Heatmap of trehalose/glycogen pathway genes expression in MSB3Δ. (F) RT‐qPCR validation of trehalose metabolism genes in WT vs. MSB3Δ (*n* = 3). (G) Trehalose/glycogen quantification in WT vs. MSB3Δ (μg/mL) (*n* = 6). (H) Autophagy‐related genes expression profiles in MSB3Δ. (I) Representative fluorescence images of ATG8‐EGFP at different time points in WT and MSB3Δ strains cultured in complete synthetic medium (2% glucose) and shift to glucose‐restricted medium (0.05% glucose). Scale bar: 5 μm. (J) Time‐lapse distribution of average ATG8‐EGFP fluorescence intensity in WT (*n* = 35) and MSB3Δ (*n* = 39) single cells grown in complete synthetic medium (2% glucose) and shift to glucose‐restricted medium (0.05% glucose). (K) Time‐lapse single cell ATG8‐EGFP fluorescence intensity dynamics in WT and MSB3Δ strains cultured in complete synthetic medium (2% glucose) and shift to glucose‐restricted medium (0.05% glucose). (L) Rapamycin sensitivity assays in WT and MSB3Δ. (M) RLS analysis of YPT6 and VPS21 deletion mutants in WT and MSB3Δ strains, with mean lifespan values indicated in the figure. All strains are in the BY4741 background. Statistical analysis was performed using GraphPad Prism v8.0 software. Data were considered statistically significant at *p* < 0.05, calculated by using Student's *t*‐test (F, G, and J) or log‐rank test (M). n.s. indicates not significant, *p < 0.05; **p < 0.01; ***p < 0.005, ****p < 0.001. All values are means ± SEM. The corresponding n values (number of cells) are shown within each sub‐plot.

MSB3 deletion downregulates many ribosome‐related genes, such as CGR1, RPL42B, RPS22A, NOP7, and RPL26B, as well as enhancing stress response genes, including TSL1, TPS1, and TPS2. PPI (protein–protein interaction) analysis shows that enhanced trehalose‐ and glycogen‐associated genes, as well as reduced ribosomal subunit maturation/export‐related genes, not only cooperate functionally, but are also physically bound (Figure 5A). Previous publications have revealed that deletion of some ribosome genes can extend yeast RLS (McCormick et al. 2015). In parallel, trehalose acts as an important reserve carbohydrate that can stabilize proteins and suppress the aggregation of denatured proteins during periods of stress (Zhang and Cao 2017). This is an established positive factor for longevity.

We also note that a group of *MSB3Δ* up‐regulated genes are functionally associated with mitochondrial metabolism or anti‐oxidative stress response (Figure 5B). Among them, ECM4 and GTT2 are both glutathione transferases, GPX1 is glutathione peroxidase, and IDH1, as well as IDH2, are mitochondria NAD (+)‐dependent isocitrate dehydrogenases, which are also involved in glutamate biosynthesis. Glutathione is an important cellular protective molecule against peroxidation, and the glutathione‐related pathway converges on many events linked to longevity (Fang et al. 2018). In addition to IDH1 and IDH2, other genes involved in mitochondrial metabolism include USV1, PIC2, AIM17, and ALD4. PIC2, for example, is a mitochondrial copper and phosphate carrier, its human homolog, SLC25A3, has been reported to be necessary for the biogenesis of cytochrome C oxidase (Boulet et al. 2018). ALD4 is a NAD (+) or NADP (+) dependent aldehyde dehydrogenase and participates in NADH regeneration (Liu et al. 2009). USV1 is a putative transcription factor that is activated in respiratory metabolism, regulating the expression of stress response genes like CTT2, ACS1, and IDH1 (Hlynialuk et al. 2008). In fact, CTT2 and ACS1 are also up‐regulated in *MSB3Δ* (data not shown, because the *p* > 0.05).

More surprisingly, we analyzed the transcriptional regulation of the aforementioned metabolic‐related genes and found they are under the regulation of two stress‐related transcription factors, SUA7 and BUR6 (Figure 5C). Furthermore, BAT2, a branched‐chain amino acid (BCAA) aminotransferase, and a Ras (Ira2) GAP (GTPase‐activating protein), TFS1, are also targets of SUA7 or BUR6. The TFS1 promoter contains two stress‐responsive elements, and its expression is elevated in response to stress. As a GAP, Tfs1p is necessary for the activity of Ira2p, and the activation of Ira2p can inhibit PKA activity, thus contributing to the expression of stress response genes, including TPS1, TPS2, et al. (Gombault et al. 2007). This analysis suggests that the MSB3 deletion leads to global down‐regulation of ribosome‐related genes, as well as up‐regulation of stress response genes. It also indicates the presence of a strong regulon that protects cells from the oxidative damage that accumulates with age in the presence of an MSB3 mutation.

### 2.6 Decreased MSB3 Attenuates the Metabolic Shift From Glycogen to Trehalose and Promotes Lifespan via Enhancing Autophagy

As trehalose biosynthetic process‐related genes are both functionally and physically enriched in up‐regulated DEGs of *MSB3Δ*, and trehalose has been previously shown to enhance the elimination of protein aggregates via autophagy, autophagy is considered to be beneficial to longevity, we maintained our focus on the trehalose metabolism (Aman et al. 2021). Figure 5D shows that UDP‐α‐D‐glucose is an intermediary for both glycogen and trehalose biosynthesis. Yeast trehalose biosynthesis is under the regulation of α‐α‐trehalose‐phosphate synthase complex, which consists of two key enzymes, TPS1 and TPS2, and two regulatory proteins, TPS3 and TSL1, whereas degradation of trehalose is catalyzed by ATH1 or NTH1. We found that, in the MSB3 deletion strain, the expression of enzymes involved in trehalose and glycogen biosynthesis was increased, as was the expression of trehalose degradation enzymes (Figure 5E). We subsequently verified the above results using RT‐qPCR. It is worth noting that the transcription of TPS1, TPS2, and GLG2 was significantly increased in BY4741 (with a *p*‐value of 0.044, 0.047, and 0.028, respectively) (Figure 5F).

Given that the above trehalose or glycogen‐related genes were all altered in the MSB3 mutant, we next examined the intracellular trehalose and glycogen levels. MSB3 deficiency resulted in a significant increase in trehalose levels in yeast cells (*p* = 0.001) (Figure 5G). Astonished, we found that decreased MSB3 contributed to a decline in glycogen level (*p* = 0.0081) (Figure 5G). Although both trehalose and glycogen are previously reported to be involved in lifespan extension, it is interesting that *Yonghak* and colleagues found that a metabolic shift from glycogen to trehalose can promote healthy longevity in *C. elegans* , indicating that there may exist a competition between biosynthesis of trehalose and glycogen in yeast; MSB3 elimination can promote the metabolic shift to trehalose storage (Honda et al. 2010; Seo et al. 2018). Further investigation is needed to delve into the underlying mechanisms in the future.

In considering the positive impact of trehalose on autophagy, we further investigated whether MSB3 deletion could interfere with autophagy. Figure 5H showed yeast autophagy‐related gene expression profiling in the MSB3 mutant. Among them, members of cargo‐recognition‐during‐selective‐macro autophagy machinery were obviously enhanced in expression, indicating a probable promotion of bulk cytoplasm or specific structure recycling. Furthermore, the Atg17‐Atg31‐Atg29 subcomplex, which is required for maximal Atg1 serine/threonine protein kinase activity, was also up‐regulated. In yeast, the Atg1 complex is thought to be involved in autophagosome formation, which is under the regulation of TORC1 (Nakatogawa et al. 2009). This suggests that the deletion of MSB3 may increase the ability of Atg1 to phosphorylate other target Atgs, thus contributing to enhanced autophagy. Besides, ATG8, a human LC3 homolog in yeast, that acts as a key molecule of autophagy initiation by organizing the phagophore assembly site (PAS), also significantly increased in the mutant.

We further tagged endogenous ATG8 with EGFP in BY4741 and checked the dynamic response of ATG8 to rapid caloric restriction stress in single cells of the MSB3 mutant and WT. Considering the long‐term cell loading operation of the microfluidics chip and the stress response property of ATG8, we changed the nutrition 4 h after cell loading, as we believed this would allow sufficient time for the cells to recover. Compared with WT, MSB3Δ significantly increased the basal expression of the endogenous cellular Atg8p. Both strains responded to a shift in nutrition from 2% to 0.05% glucose by upregulating Atg8p (Figure 5I–K). As shown in Figure 5I–K, whether in WT or MSB3Δ, cellular Atg8p slightly decreased after cell loading in a time‐dependent manner, and most of them returned to a similar level 3 h after cell loading. The shift of glucose awoke a significantly strong response of Atg8p in both single cells and bulk cells of MSB3Δ, suggesting that deprivation of MSB3 leads to stronger responsiveness.

We also tested the response of cells to rapamycin stimulation, as shown in Figure 5L, rapamycin suppressed cell growth of the two strains equally, indicating the enhanced stress response capability of the MSB3 mutant is mTOR‐independent.

### 2.7 The Involvement of MSB3 in Longevity Is Associated With Its Function as a GAP

MSB3 is a Rab GTPase‐activating protein that is responsible for regulating endocytosis, as well as vacuole fusion and transport. In general, the absence of a GAP will result in long‐term, low‐level Rab activation. As a GAP, MSB3 regulates GTPases, including VPS21, YPT6, SEC4, YPT1 and YPT31/32 (Figure S6 shows the relationship between these genes and their expression in MSB3 deletion). Among them, the first two, VPS21 and YPT6, are the primary targets of MSB3 and autophagy‐related (Nakatogawa et al. 2009). Consequently, we tested the possibility that a double mutant of VPS21 or YPT6 in MSB3Δ could attenuate the longevity phenotype of MSB3Δ. As shown in Figure 5M, MSB3Δ significantly increased RLS of yeast, VPS21 deletion showed reduced lifespan, and double deletion of VPS21 and MSB3 exhibited a similar lifespan to VPS21Δ, suggesting the utmost importance of VPS21 to yeast lifespan maintenance, and the longevity phenotype of MSB3Δ may be VPS21 independent. On the contrary, a single mutation of YPT6 showed minimal impact on yeast RLS, while double deletion of YPT6 and MSB3 could eliminate the positive contribution of MSB3Δ to longevity. The above results indicate that the involvement of MSB3 in longevity is partly related to its function as a GAP of Rab GTPase, especially YPT6.

## 3 Discussion

Genetic analysis of complex human traits (e.g., GWAS of human height) typically identifies a large number of variations associated with the phenotype; these variations are distributed across the genome, mapping to many genes with seemingly unrelated functions, making it very challenging to delineate the mechanism. Motivated by the observation, Boyle et al. proposed the “omnigenic” model which states that there is a set of “core genes” mechanistically connected to the phenotype, and there are many “peripheral genes” that can influence the phenotype by interacting with the core genes through a complex gene regulatory network (Boyle et al. 2017). Inspired by the omnigenic model, we extended the framework to propose a three‐layered network architecture with a fan‐in and fan‐out structure: a large set of “peripheral genes” connected to a much smaller set of “master regulators” which then regulate a larger set of “core genes” organized into functional modules to influence the phenotype (Figure 1). Using yeast replicative aging as an example of complex phenotypes, and by combining mutant phenotype and gene expression data, we developed a mathematical approach to (1) identify master regulators, (2) define peripheral genes and connect them to the MRs, and (3) delineate downstream functional modules of core genes that are connected to the MRs. We showed that such an approach allows us to identify novel lifespan extending perturbations with high accuracy (Figure 3), and to discern downstream functional modules that consistently respond to lifespan extending perturbations (Figure 4).

It is worth noting that although we only used non‐essential gene deletion mutant data to develop the model, we were able to predict the lifespan effect of over‐expression and down‐regulation of essential genes, indicating the power of the generalization of the theoretical model.

How realistic is the three‐layered network architecture for complex traits? In the context of aging, the existence of master regulators has long been realized. One classic example is Daf2/Daf16 for regulating the lifespan of worms (and the insulin/IGF signaling for mammalian species) (Kenyon 2010). Mutation of Daf2 or constitutive activation of Daf16 (a transcription factor downstream) significantly extends lifespan, and many other lifespan extending mutants function through/depend on Daf16 (Zhao et al. 2021; Kenyon et al. 1993; Li et al. 2021). Daf16 regulates hundreds of genes downstream in concert to exert a strong lifespan effect, while each of the target genes only contributes a small effect (Murphy et al. 2003). Thus, this is an example of a three‐layered fan‐in and fan‐out structure. TOR pathway is another example: many lifespan extending perturbations depend on the down‐regulation of TOR, and TOR coordinately regulates many functional classes of genes (such as protein synthesis and autophagy) to influence lifespan (Chin et al. 2014; Mannick and Lamming 2023).

In our model in which gene expression change contributes additively to the lifespan change, the MRs have a number of special properties: (1) they are features selected by the LASSO regression model, indicating that their expression change contributes to the lifespan change of multiple mutants; (2) consistent with this interpretation, simple statistical analyses indicate that the MRs have stronger response in mutants with increased lifespan and their directions of change are more consistent across the mutants (Figure 2E,F); (3) our subsequent experimental tests also showed that perturbing the MRs in the predicted direction is much more likely to lead to lifespan extension than genes picked at random. Furthermore, functional analyses seem to suggest that the MRs are placed at the central hubs of the gene regulatory network, such as Sir2 and Top2 for genome/epigenome maintenance, Sul1 for signaling to PKA, and molecular chaperons for stress response and protein quality control. Together, these pieces of evidence suggest that MRs identified by the math model indeed occupy special positions in the gene regulatory network that controls lifespan.

We should note the our current definition of MR vs. PG are mostly mathematical, based on their properties from the Lasso regression model. To better validate this concept and understand the distinction between MRs and PGs, we need better biological definitions. In the context of aging, MRs are the genes that can coherently regulate a large set of downstream genes to achieve lifespan extension, and they also mediate the lifespan effect of many other genes (e.g., DAF16 in worm, and SIR2 in yeast). PGs are those whose lifespan phenotype is mediated through MRs and mathematically we define them as those whose lifespan phenotype can be explained by the MRs they are connected to. Ideally if we have the complete network for aging mapped out (which we don't), we can define MRs as the converging points of lifespan extending perturbations with a high number of incoming arrows and outgoing arrows (fan in and fan out). We hope that in the future as more accurate and comprehensive gene regulatory network for aging is constructed, we can use this more biological definition in conjunction with the mathematical definition to better delineate MRs and PGs.

We used TF modules as proxy for functional modules for our analysis, since our work is mainly about connecting transcriptional profile to lifespan (which has its limitations as we discuss below). We feel that using transcription factor module is a natural and unbiased way to analyze the functional changes due to genetic perturbation (e.g., deletion or over‐expression of a gene). Using all the targets of a TF to analyze the response is a natural way to perform dimensional reduction and to average out the noise (gene expression data is noisy at the individual gene level). In addition, we have good information to construct transcription factor modules in yeast due to the availability of systematic ChIP‐chip data (Lee et al. 2002; Wang et al. 2005). Many of the downstream modules implicated (stress response, proteostasis, mitochondrial function, translation, etc.) are well established in the aging field, but we identified them from an unbiased approach, without prior assumptions, indicating that using TF module to analyze different longevity mutants can identify a set of conserved functional requirements for lifespan extension.

It is interesting to contrast our approach with existing network models of aging. Traditionally, influential nodes in biological networks have been identified based on topological properties, such as high connectivity or centrality within protein–protein interaction networks (Vural et al. 2014; Qin 2019). Several studies analyzed the relationship between network structure and statistical properties of lifespan distribution. For instance, Vural et al. demonstrated that the qualitative features of aging, such as the exponential increase in mortality rate, are remarkably robust to network topology, emerging from both random and scale‐free dependency networks (Vural et al. 2014). Qin's parsimonious model, grounded in reliability theory, interprets aging through the decay of gene interactions, estimating parameters like the average number of lifespan‐influencing interactions per essential node (n) without explicitly defining a hierarchical regulatory structure (Qin 2019). These approaches, often categorized as “network map” models, are powerful for describing the global architecture and statistical properties of aging systems but are not designed to pinpoint specific, experimentally actionable genes that orchestrate the aging process (Su and Hao 2025). In contrast, we define MRs functionally, based on their quantitative contribution to lifespan variation across a large panel of genetic perturbations. This approach aligns more closely with the “dynamical models” discussed in a recent review, which focus on simulating the temporal behavior of interacting factors to gain mechanistic insights (Su and Hao 2025). Our method identifies MRs as the key nodes that connect peripheral genes to core functional modules, predicting not only which genes are important but also the direction of their perturbation (up‐ or down‐regulation) to extend lifespan. Thus, while previous network models have successfully explained how aging emerges from system properties (Vural et al. 2014; Qin 2019), our approach identifies specific master regulators of lifespan, providing a more targeted and experimentally testable framework for understanding cellular aging.

To situate our findings in the context of established paradigms in yeast aging research, we also examined the relationships between the identified MRs/downstream core modules and the canonical aging pathways. Studies on yeast replicative aging have revealed several key regulatory axes, including genome stability maintenance mediated by silent information regulators (e.g., SIR2) (Hughes and Gottschling 2012), TOR signaling and ribosome biogenesis (Hughes and Gottschling 2012; Kaeberlein et al. 2005), mitochondrial respiration and retrograde signaling (Janssens et al. 2015; Li et al. 2017), and the more recently proposed protein homeostasis network (Li et al. 2017; Zou et al. 2020; Li et al. 2020). Our study implicated these classical pathways without any prior assumption and offered a network‐level interpretation for them. Transcription factor module analysis revealed that multiple longevity‐associated perturbations—including deletions of MSB3, NIT3, HSP26, and the TYS1‐DAmP allele—consistently induced significant downregulation of ribosomal genes (Figure 4D). This transcriptional signature mirrors the ribosomal repression observed in canonical long‐lived mutants such as tor1Δ and sch9Δ, reinforcing the general observation that reduced translational capacity promotes lifespan extension (Kaeberlein et al. 2005). Concurrently, these lifespan extending perturbations upregulated stress‐responsive modules (enriched for Msn2/4 target genes) and respiration‐associated gene sets, consistent with the model that the balance between mitochondrial and ribosomal expression governs aging trajectories (Janssens et al. 2015). Interestingly, SIR2 overexpression elicited an opposite transcriptional profile (Figure 4C), suggesting that its pro‐longevity effects operate through distinct mechanisms—likely involving heterochromatin maintenance and rDNA stabilization—rather than through the ribosomal‐mitochondrial balance observed in other contexts (Sinclair and Guarente 1997).

Our TF module analysis of MR perturbations also uncovered a striking pattern: despite minimal gene‐level overlap (with CGR1 being the sole commonly downregulated transcript), the downstream functional modules affected—including ribosome, proteasome, mitochondrial genes, cell cycle regulators, and amino acid metabolic pathways—exhibited highly concordant responses across perturbations (Figure 4C,D). This “upstream regulatory divergence with downstream functional convergence” represents a key organizational principle of the aging transcriptional network, consistent with proposal that aging is governed by core functional modules rather than individual genes (Hughes and Gottschling 2012).

We note that there are exceptions to the general picture described above. Some of the MRs identified by the model may be directly linked to lifespan mechanistically, thus by definition can also be a core gene, if that gene is involved in a process that is rate‐limiting for the lifespan. For example, two of the MRs identified by the model are TSA1 (Thioredoxin peroxidase), which is an antioxidant, and CTS1, which is an Endochitinase required for cell separation after mitosis. We hypothesize that increased oxidative stress and decreased ability for daughter cell separation could be rate‐limiting for lifespan, thus improvement in these aspects is required in general for lifespan extension. This will be reflected in consistent gene expression change across long‐lived mutants, and thus be picked up by the model.

Why didn't the model pick up many core genes as MRs, given that lifespan extending mutants seem to converge to a set of functional core modules with consistent direction of change? We argue that the convergent transcriptional program is only at the level of transcription modules, not at individual genes, i.e., there are different solutions to improve the function of a TF module. Thus, a single gene does not always respond to lifespan‐extending mutations. In addition, lifespan extension may require core genes to act cooperatively; thus, the change of a single core gene is not sufficient to produce lifespan extension. A single core gene change in general is neither necessary nor sufficient for lifespan extension, which may lead to a weak correlation with lifespan phenotype.

There are a number of limitations of the model we proposed. First, we use a transcriptional profile to approximate gene expression, therefore, we will miss genes with important post‐transcriptional and post‐translational regulation. One such example is TOR1, which is known to be a master regulator for lifespan, but was missed in our LASSO regression model. It is known that TOR activity is regulated through interaction with small GTPases, scaffolds, and nutrient sensors. We suspect that in many lifespan‐extending mutants, TOR activity may go down, but not at the transcript level. Our model may also generate other false positives and false negatives, given a limited number of mutants with both lifespan and gene expression data (and only ¼ of ~200 longevity mutants), thus a small training set to train the model. We expect the performance of the model will improve as more mutant lifespan and gene expression data become available.

We believe the three‐layered architecture with peripheral genes, MRs, and core functional modules with a fan‐in, fan‐out structure may exist for complex human traits. We suggest that similar mathematical approaches that combine genetic data on phenotypes and gene expression may be applied. For example, GWAS data may be considered genetic perturbation (through natural variation) vs. phenotype data, and eQTL data can be considered as genetic perturbation vs. gene expression data. With the rapid accumulation of tissue‐specific eQTL data and enhancer mapping data, our mathematical framework may lead to new insight into the master regulators and functional modules for complex human traits.

## 4 Materials and Methods

### 4.1 Database for LASSO Model Construction

Two datasets were utilized for the construction and analysis of the LASSO model in this study. The first dataset comprises gene expression profiles of 1479 mutants (GSE42528). The second dataset includes yeast replicative lifespan data for knockout strains, which measures the percentage change in lifespan and the number of cells (McCormick et al. 2015).

### 4.2 LASSO Model Cross‐Validation

As an additional validation of the LASSO model, “keep‐one‐out” validation was employed to predict lifespan. It was performed as follows: one sample served as the testing set while all other samples constituted the training set. This approach yielded a predicted lifespan for each mutant without utilizing the mutant itself for prediction (Figure S2C,D). In this validation framework, the lifespan is not directly influenced by the expression of the gene; however, it can still be predicted based on variations in gene expression that are responsive to other gene mutations. The data points should be randomly distributed across the entire figure, and the results demonstrate a strong linear relationship.

### 4.3 Yeast Strains and Plasmids

*Saccharomyces cerevisiae* strain, BY4741 (*MATa his3Δ1 leu2Δ0 met15Δ0 ura3Δ0*) and BY4742 (*MATalpha his3Δ1 leu2Δ0 lys2Δ0 ura3Δ0*) and their deletion derivatives used in the study are mentioned in Table S9. The list of plasmids used for the generation of the deletion marker cassette to replace ORF and the sequences of primers used for PCR are mentioned in Table S9.

Yeast cultures were cultivated in YPD complete medium containing 2% glucose, 2% peptone, 1% yeast extract, or synthetic complete medium supplemented with 0.03% essential amino acids and 2% glucose. The glucose‐restriction media contained the same components as the complete medium except for a reduced concentration of glucose at 0.05%. Yeast strains were grown at 30°C and shaken at 250–300 rpm.

Knockout strains were generated by transforming with a PCR product encoding the kanamycin resistance (KanR) cassette or HisMX6 cassette to replace the ORF of interest. EGFP‐labeled strains were generated by inserting an EGFP‐HisMX6 cassette amplified from the pYM‐N18‐GFP‐HisMX6 plasmid into the C‐terminal of the target gene.

TOP2, TYS1, and ERg25 DAmP allele construction was performed as previously described (Breslow et al. 2008). In brief, the kanamycin‐resistance (KanR) cassette was inserted immediately downstream of the open reading frame of the target gene via transformation with a PCR product containing the KanR cassette, which was flanked at both ends by homologous sequences to the targeted locus. This disrupted the stability of the corresponding transcript and typically reduced the amount of mRNA.

We employed two strategies to construct overexpression strains. The first strategy involved inserting a single‐copy PCR‐amplified product of the target gene, along with a kanamycin resistance (KanR) cassette, into the Leu deletion region. This insertion resulted in an approximately twofold overexpression of the target gene. The second strategy replaced the native promoter with the stronger TEF1 promoter by inserting the TEF1 promoter and a selectable marker upstream of the target gene's open reading frame (ORF). This approach significantly enhanced target gene expression, although the exact level of overexpression was variable and not tightly regulated.

### 4.4 Life Span Analysis

Replicative lifespan analysis by microdissection and single‐cell tracking using the microfluidic device was performed as previously described. Micromanipulation dissections were performed at laboratory temperature. The microfluidic chip for yeast was kindly provided by Prof Chunxiong Luo of PKU.

### 4.5 RNA‐Seq and Differential Expression Gene Analysis

Yeast in the logarithmic growth phase (∼0.5 OD_600_) was harvested, and RNA‐seq experiments were conducted following the previously described protocol on BGISEQ‐500 (Beijing Genomics Institute). The sequencing data discussed in this publication have been deposited with the NCBI Gene Expression Omnibus and are accessible through GEO Series accession number GSE305491 (http://www.ncbi.nlm.nih.gov/geo/query/acc.cgi?acc= GSE305491). The gene expression levels across different samples were compared using Fragments Per Kilobase Million (FPKM) values. The log2 ratio of the FPKM values was employed to calculate the fold change in gene expression for each sample. Subsequently, R software was utilized to perform a differential expression analysis between the samples (Reuter et al. 2015).

### 4.6 RNA Extraction, Reverse Transcription and Real‐Time qPCR

Total RNA was extracted using TRIzol reagent (Invitrogen, #15596026) following the manufacturer's instructions. Residual DNA was removed using the gDNA Wiper Mix, and 1 μg of total RNA was reverse‐transcribed into cDNA with HiFiScript gDNA Removal RT MasterMix (CWBIO, Jiangsu, China). Quantitative real‐time PCR was performed on the 7500 Fast Real‐Time PCR System (Applied Biosystems, Foster City, CA, USA) using 2× SuperFast Universal SYBR Master Mix (CWBIO, Jiangsu, China). Actin was used as the internal control for data analysis. The relative expression levels of mRNA were calculated using the 2 − ΔΔCT method. Each measurement included three technical replicates. All primer sequences are included in Table S9.

### 4.7 Transcriptional Factors Prediction of DEGs

We employed the regulator function module of the Saccharomyces Genome Database (SGD) to identify transcription factors associated with differentially expressed genes (DEGs) in the MSB3Δ transcriptome sequencing data and to rank their frequency. Transcription factors that exhibited higher frequencies were deemed more likely to serve as common regulators of DEGs.

### 4.8 Calculation of the Contribution of Predicted MRs in the RLS Extension in Long‐Lived Peripheral Gene Mutants

Pick up longevity mutants from Two datasets (RLS extension ≥ 15%, and *p* < 0.05, and have gene expression profiling data); then pick up mutants being well predicted by LASSO model (selection criteria: |L1‐L2|/(|L1| + |L2|)< 0.3 or 0.5 or 0.7; L1 = real RLS, L2 = predicted RLS); for each well predicted mutant, we then calculate *x*_*ij*_ *β*_*j*_ of each gene in the whole genome, and rank contributed genes according to *x*_*ij*_ *β*_*j*_; for each well predicted mutant, we choose its Top5 or 10 or 20 contributed genes, and constructs the interactions.

### 4.9 Module Score (*Z*) Calculation

The module score is the *z*‐score from a Mann–Whitney *U* test on the set of genes that are regulated by a transcription factor (based on ChIP‐chip experiment) against all the other genes. For a given differential expression gene list (a gene expression fold change comparing a mutant strain vs. a wildtype strain), and a given module (genes regulated by a specific TF), we calculated the rank sum of the genes in the module and genes in the background set, then computed the *z*‐score and corresponding *p*‐values. The module score summarizes how strongly a TF's targets are collectively shifted in the differential expression ranking relative to non‐targets. Larger *Z* values indicate stronger coordinated regulation consistent with the contrast.

SUL1Δ, MSB3Δ, NIT3Δ, HSP26Δ, TYS1 DAmP profiling data were from our sequencing data, Sir2OE profiling data were collected from (https://www.ncbi.nlm.nih.gov/geo/query/acc.cgi?acc=GSE142864), which included RNA profiling data of 9 timepoints (SIR2_t0, SIR2_t5, SIR2_t30, etc.). TFs modules which had a|*Z* scores|> 2.5 were chosen as significantly changed modules. To briefly characterize TFs module score of SIR2OE, for each module, we calculated the mean score of three timepoint SIR2OE data (SIR2_t30, SIR2_t45, SIR2_t90). In the TFs module analysis, we always annotate to some modules of the same name, which generally correlate with different growth conditions. To concisely illustrate the regulation, in the heatmap, the same TF modules were combined by calculating the mean scores. And we employed hierarchical clustering to do the clustering.

### 4.10 Rank the Mean Expression of Ribosome Genes as Well as Other Genes of the Whole Genome in Longevity Mutants

The list of ribosomal genes was obtained from the Saccharomyces Genome Database (https://www.yeastgenome.org/search?category=locus&cellular_component=ribosome%20%28direct%29&geneMode=wrap&page=0&q=Ribosomal%20Protein). After excluding mitochondrial genes, 137 ribosomal genes remained. Among them, 127 genes with gene expression profiling data were selected for analysis. For each gene, we calculated the mean expression in longevity mutants. Then we conducted a rank test comparing the mean expression of ribosomal genes to that of control genes in these longevity mutants. Additionally, for each longevity mutant, we compared the expression of ribosomal genes to that of control genes within the same mutant and computed a rank test *z*‐score for each mutant.

### 4.11 Metascape Gene List Analysis for PPI (Protein–Protein Interactions) Analysis

DEGs of MSB3 deletion data were submitted to Metascape (https://metascape.org), and PPI analysis was done automatically. For PPI analysis, all protein–protein interactions among input genes were extracted from the PPI data source and formed a PPI network (Zhou et al. 2019).

### 4.12 Trehalose Storage and Glycogen Storage

A single yeast clone was inoculated in 5 mL of YPD medium and incubated overnight at 30°C with shaking at 200 rpm. Following dilution to a concentration of OD600 = 0.1, the culture was incubated for 4 h under the same conditions until it reached the exponential growth phase. The cells were washed with 1 mL of sterile ice water, and the cell concentration was quantified. A total of 1 × 10^8^ cells were transferred to a 96‐well plate, centrifuged, and the supernatant was subsequently removed. After being resuspended in 125 μL of Na_2_CO_3_, the cells were incubated at 95°C for 3 h with occasional rotation. The plates were then cooled to room temperature, and the cell suspension was thoroughly mixed before being evenly divided into two new plates. Three technical replicates were performed for each strain.

For glycogen measurements, each reaction solution was prepared by combining 188 μL of amyloglucosidase buffer, 62 μL of cell suspension, and 10 μL of freshly prepared Aspergillus niger α‐amyloglucosidase solution (approximately 70 U/mg) in a sodium acetate buffer (pH 5.2) at a concentration of 0.2 M. The plates were mixed thoroughly and incubated overnight at 57°C. For trehalose measurements, each reaction solution was prepared by adding 188 μL of trehalase buffer, 62 μL of cell suspension, and 10 μL of porcine trehalase solution (approximately 0.007 U) that was diluted threefold with 0.2 M sodium acetate buffer (pH 5.2). The plates were then incubated overnight at 37°C. The quantification of glucose released during these procedures was performed using the Glucose Assay Kit (Sigma‐Aldrich, #MAK263) according to the manufacturer's instructions.

### 4.13 Single‐Cell Time‐Lapse Imaging

The cells were cultured overnight in synthetic complete media containing 2% glucose at 30°C until they reached an optical density of approximately 1.0. Subsequently, the cells were diluted tenfold and further cultivated in a shaker at 30°C for an additional 4–6 h before being loaded into the microfluidic device. The microfluidic device was employed to track single cells, following the previously described protocols (Xie et al. 2012; Chen et al. 2020). The microfluidic chip used in this experiment was generously provided by Professor Chunxiong Luo from Peking University.

### 4.14 Rapamycin Sensitivity Experiment

In the experimental group, a rapamycin solution was added to the YPD medium to achieve a final concentration of 10 nM, while an equivalent volume of DMSO was added to the control group. The strains were inoculated in YPD liquid medium and cultured overnight. The following day, the culture was diluted to an optical density (OD600 = 0.1) and incubated for 4 h at 30°C with shaking at 200 rpm to maintain the cells in the exponential growth phase. The culture was then further diluted to an OD600 of 0.1 and subjected to five serial fivefold dilutions. Subsequently, 1 μL of each dilution was inoculated into separate rapamycin and DMSO medium plates. The plates were incubated at 30°C, and growth was recorded every 12 h. ImageJ software was used to analyze the growth curves of both the experimental and control groups.

### 4.15 Statistical Analysis

The replicative lifespan was analyzed using the Kaplan–Meier method, and survival differences were assessed via the log‐rank test. The Kolmogorov–Smirnov test was used to evaluate the distribution differences between two groups. Group differences were further evaluated using Student's *t*‐test. All statistical analyses were conducted using SPSS (version 22.0) and GraphPad Prism (version 8). A *p* < 0.05 was considered statistically significant. To evaluate statistical significance both between and within groups, a one‐way ANOVA followed by post hoc Bonferroni analysis was applied.

## Author Contributions

J.Y., H.L. and Y.Z. conceived and designed the experiments. M.M. J.L., Y.C., and M.Z. performed the yeast experiments. Y.S., H.L. and J.Z. performed the LASSO model. J.Y., M.K. and B.K.K. provided lifespan data for LASSO Regression. M.M., J.L., Y.C., Y.X. and L.Y. performed the bioinformatics analysis. J.Y., H.L. wrote the manuscript with input from all authors. All authors read and approved the final manuscript.

## Funding

This work was supported by Sichuan Province Science and Technology Support Program (2023YFS0050, 2024YFHZ0009).

## Consent

The authors have nothing to report.

## Conflicts of Interest

The authors declare no conflicts of interest.

## Supporting information


**Figure S1:** Sensitivity analysis of the weighting constant in LASSO feature selection. Venn diagrams showing the overlap of master regulator (MR) genes identified under three different weighting constants (30, 50, and 80) in different models. (A) MR genes identified as the overlap between the non‐linear and “self0” models. (B) MR genes identified using the non‐linear model. (C) MR genes identified using the “self0” model. The substantial overlap across the three weighting constants demonstrates that the selection of MR genes is robust to the choice of the stabilizing constant, supporting the reliability of the identified master regulators.
**Figure S2:** Model training and evaluation. (A) Determination of the penalty parameter α in the “self0” model via cross‐validation. (B) Evaluation of the model prediction efficiency by comparing the observed and predicted RLS changes in mutant strains (self0 model). (C) keep‐one‐out cross‐validation and evaluation of the prediction efficiency of the model (self0 model). (D) keep‐one‐out cross‐validation and evaluation of the prediction efficiency of the model (non‐linear model). (E) Average magnitude of the expression change: MR genes vs. other genes across long‐lived mutants (self0 model). (F) Directional consistency of expression changes: MR genes vs. other genes across long‐lived mutants (self0 model). (G) Ratio of directional consistency to average magnitude of expression change for MR genes vs. other genes (self0 model).
**Figure S3:** qPCR validation of gene expression and dose‐dependent lifespan extension mediated by SIR2 and RHO5. (A) qPCR validation of the expression levels of TOP2 and TYS1 genes in TOP2 DAmP and TYS1 DAmP strains, respectively. (B) qPCR validation of the expression levels of SIR2 and RHO5 genes in SIR2 over and RHO5 over strains, respectively. These strains were constructed by integrating an additional gene copy at the LEU2 locus. (C) qPCR validation of the expression levels of SIR2 and RHO5 genes, and experimental measurement of the lifespan in SIR2 over and RHO5 over strains. These strains were constructed by replacing the native promoter with the constitutive TEF1 promoter. Data were considered statistically significant at p < 0.05, calculated by using Student's t‐test, all values are means ± SEM. *p < 0.05; **p < 0.01; ***p < 0.005.
**Figure S4:** Contribution of master regulators (MRs) to replicative lifespan (RLS) extension in long‐lived peripheral gene (PG) mutants for evaluating PG–MR regulatory relationships. The analysis assesses the extent to which MR expression changes contribute to lifespan extension in PG deletion strains that exhibit prolonged RLS. Panels (A), (B), and (C) correspond to contribution thresholds of 0.3, 0.5, and 0.7, respectively. And top10 contributing MRs for each PG were chosen, representing the minimum contribution score required to classify a PG–MR relationship as functionally relevant. For each threshold, the figure displays the proportion of long‐lived PG mutants in which the identified MRs show consistent expression changes associated with RLS extension, thereby providing evidence for regulatory interactions between PGs and MRs. The use of multiple thresholds demonstrates the robustness of the observed PG–MR associations across varying stringency levels.
**Figure S5:** Ribosome gene expression is commonly downregulated in longevity mutants. Expression levels of all genes in the whole genome were ranked in all longevity mutants (see Section 4). The expression level of ribosomal genes was significantly lower compared to the control group. *Z*‐score analysis for each mutant shows a normal distribution, indicating that modulation of ribosome pathways is a common feature of longevity.
**Figure S6:** Regulatory relationship and expression of MSB3 and its related GTPases. (A) Schematic diagram of the regulatory relationship between MSB3 (a GAP) and its associated GTPases. (B) Expression profiles of the enzymes involved in the MSB3‐related GTPase regulatory network.

**Table S1:** The coefficient values regressed by “self0” or “non‐linear” for each gene selected by Lasso.

**Table S2:** Top 5, 10, and 20 genes with the largest contributions for each accurately predicted mutant based on the nonlinear model (threshold = 0.3).

**Table S3:** Top 5, 10, and 20 genes with the largest contributions for each accurately predicted mutant based on the nonlinear model (threshold = 0.5).

**Table S4:** Top 5, 10, and 20 genes with the largest contributions for each accurately predicted mutant based on the nonlinear model (threshold = 0.7).

**Table S5:** Transcription profiling of MSB3Δ.

**Table S6:** Transcription profiling of NIT3Δ.

**Table S7:** Transcription profiling of HSP26Δ.

**Table S8:** Transcription profiling of TYS1 DAmP.

**Table S9:** Yeast strains & Plasmid & Primers used for this work.

## Data Availability

The data that support the findings of this study are available in the Supporting Information of this article.
