# Supplementary material for: Inferring Gene Regulatory Network Architecture Underlying Complex Traits: An Integrative Analysis of Mutant Lifespan and Gene Expression Profiles Identifies Master Regulators and Key Functional Modules for Yeast Aging
Source: Aging Cell. 2026 Apr 25;25(5):e70511. doi: 10.1111/acel.70511 (PMC13109641; doi:10.1111/acel.70511)
Supplement: Supplementary file 1 — Figure S1: Sensitivity analysis of the weighting constant in LASSO feature selection. Venn diagrams showing the overlap of master regulator (MR) genes identified under three different weighting constants (30, 50, and 80) in different models. (A) MR genes identified as the overlap between the non‐linear and “self0” models. (B) MR genes identified using the non‐linear model. (C) MR genes identified using the “self0” model. The substantial overlap across the three weighting constants demonstrates that the selection of MR genes is robust to the choice of the stabilizing constant, supporting the reliability of the identified master regulators. Figure S2: Model training and evaluation. (A) Determination of the penalty parameter α in the “self0” model via cross‐validation. (B) Evaluation of the model prediction efficiency by comparing the observed and predicted RLS changes in mutant strains (self0 model). (C) keep‐one‐out cross‐validation and evaluation of the prediction efficiency of the model (self0 model). (D) keep‐one‐out cross‐validation and evaluation of the prediction efficiency of the model (non‐linear model). (E) Average magnitude of the expression change: MR genes vs. other genes across long‐lived mutants (self0 model). (F) Directional consistency of expression changes: MR genes vs. other genes across long‐lived mutants (self0 model). (G) Ratio of directional consistency to average magnitude of expression change for MR genes vs. other genes (self0 model). Figure S3: qPCR validation of gene expression and dose‐dependent lifespan extension mediated by SIR2 and RHO5. (A) qPCR validation of the expression levels of TOP2 and TYS1 genes in TOP2 DAmP and TYS1 DAmP strains, respectively. (B) qPCR validation of the expression levels of SIR2 and RHO5 genes in SIR2 over and RHO5 over strains, respectively. These strains were constructed by integrating an additional gene copy at the LEU2 locus. (C) qPCR validation of the expression levels of SIR2 and RHO5 genes, and experime [file ACEL-25-e70511-s005.docx]

**Supplementary Figure 1**


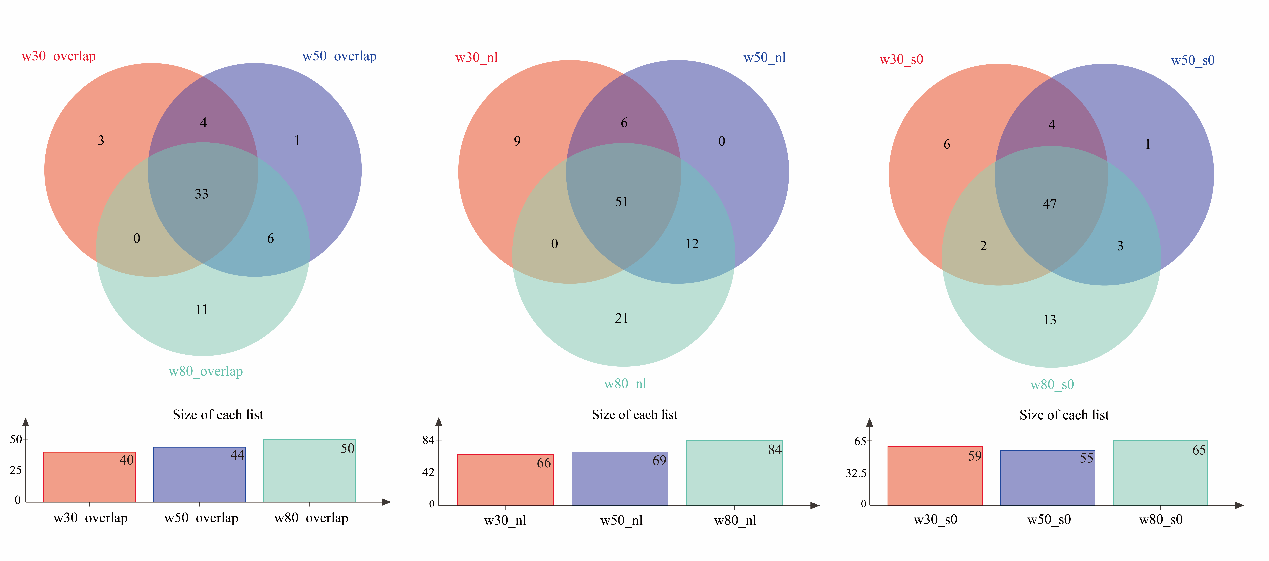


**Supplementary Figure 1**. **Sensitivity analysis of the weighting constant in LASSO feature selection.**

Venn diagrams showing the overlap of master regulator (MR) genes identified under three different weighting constants (30, 50, and 80) in different models. (A) MR genes identified as the overlap between the non-linear and "self0" models. (B) MR genes identified using the non-linear model. (C) MR genes identified using the "self0" model. The substantial overlap across the three weighting constants demonstrates that the selection of MR genes is robust to the choice of the stabilizing constant, supporting the reliability of the identified master regulators.


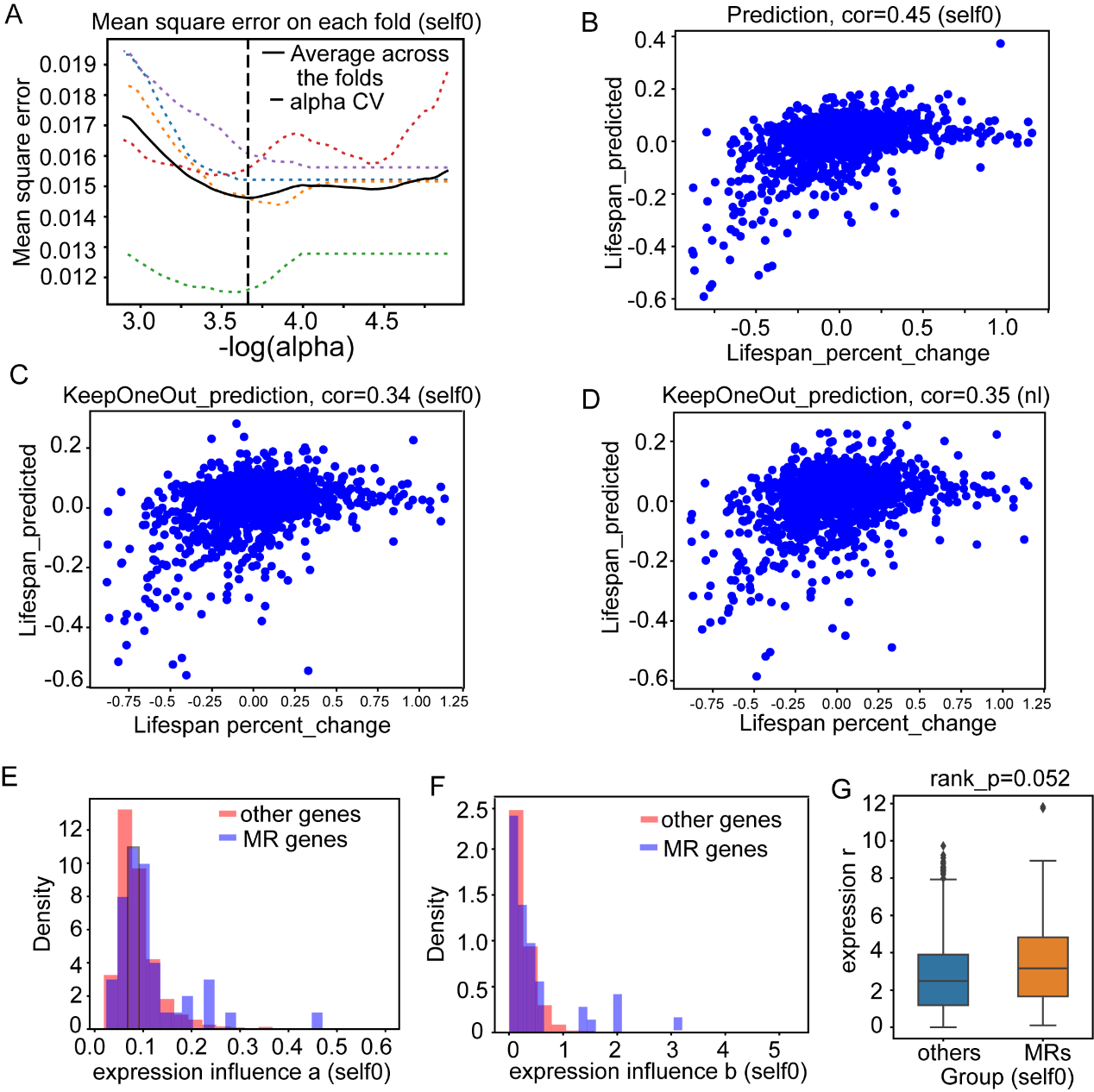
**Supplementary Figure 2**

**Supplementary Figure 2**. **Model training and evaluation**

(A) Determination of the penalty parameter α in the "self0" model via cross-validation. (B) Evaluation of the model prediction efficiency by comparing the observed and predicted RLS changes in mutant strains (self0 model). (C) keep-one-out cross-validation and evaluation of the prediction efficiency of the model (self0 model). (D) keep-one-out cross-validation and evaluation of the prediction efficiency of the model (non-linear model). (E) Average magnitude of the expression change: MR genes vs. other genes across long-lived mutants (self0 model). (F) Directional consistency of expression changes: MR genes vs. other genes across long-lived mutants (self0 model). (G) Ratio of directional consistency to average magnitude of expression change for MR genes vs. other genes (self0 model).

**Supplementary Figure 3**


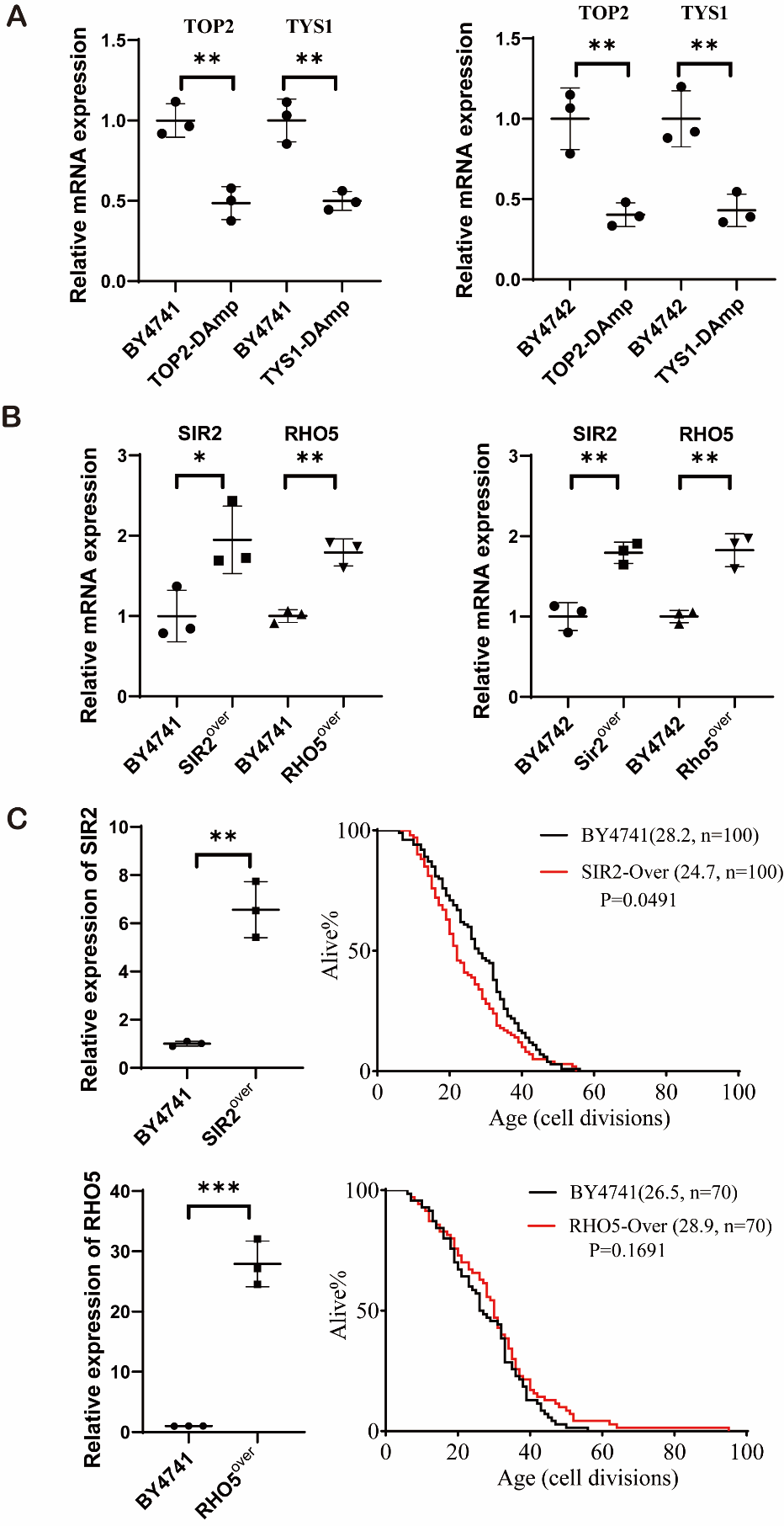


**Supplementary Figure 3. qPCR validation of gene expression and dose-dependent lifespan extension mediated by SIR2 and RHO5**

(A) qPCR validation of the expression levels of TOP2 and TYS1 genes in TOP2 DAmP and TYS1 DAmP strains, respectively. (B) qPCR validation of the expression levels of SIR2 and RHO5 genes in SIR2 over and RHO5 over strains, respectively. These strains were constructed by integrating an additional gene copy at the LEU2 locus. (C) qPCR validation of the expression levels of SIR2 and RHO5 genes, and experimental measurement of the lifespan in SIR2 over and RHO5 over strains. These strains were constructed by replacing the native promoter with the constitutive TEF1 promoter. Data were considered statistically significant at p < 0.05, calculated by using Student's t-test, all values are means ± SEM. * p < 0.05; ** p < 0.01; ***p < 0.005.

**Supplementary Figure 4**


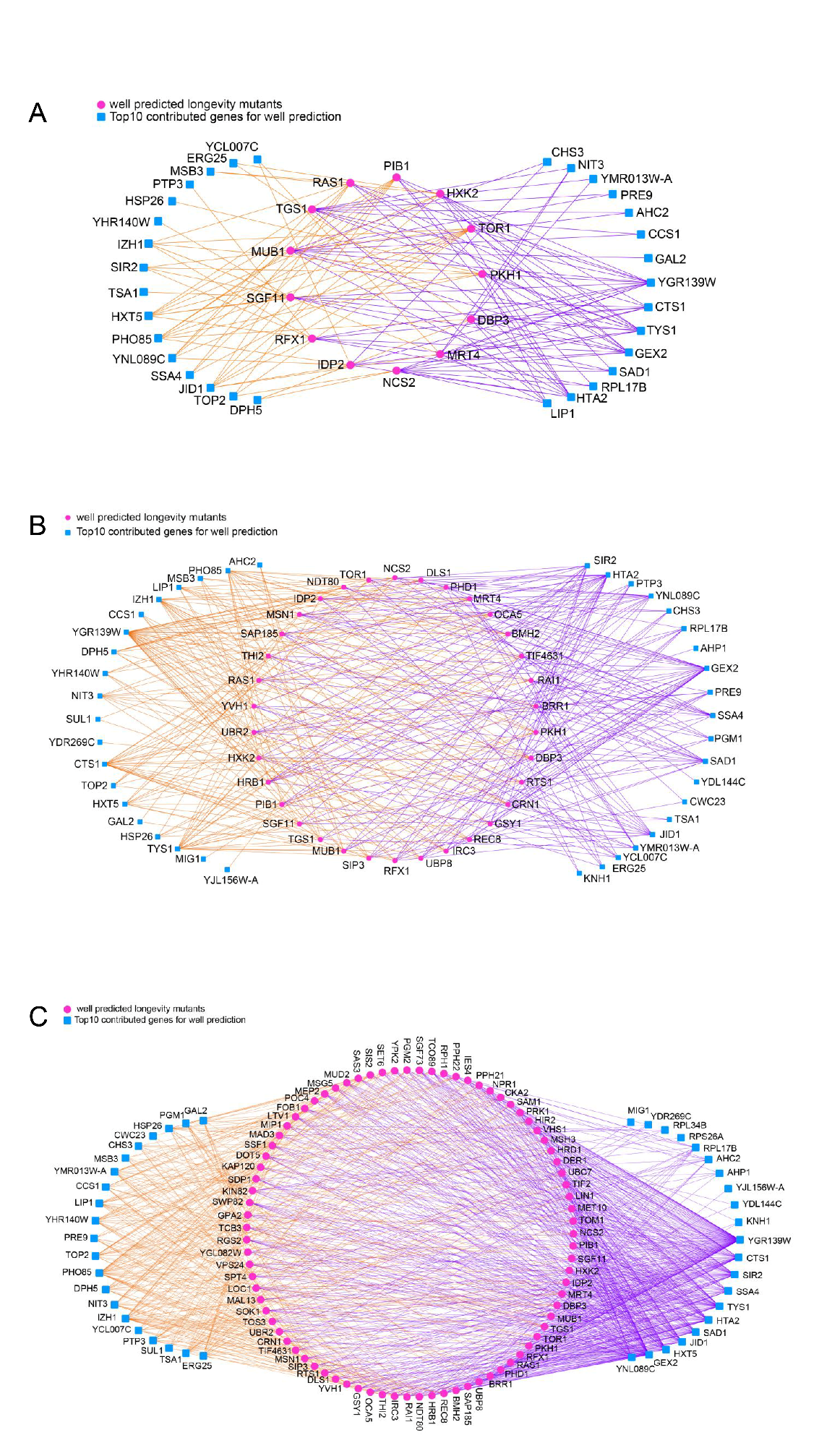


**Supplementary Figure 4. Contribution of master regulators (MRs) to replicative lifespan (RLS) extension in long-lived peripheral gene (PG) mutants for evaluating PG–MR regulatory relationships**

The analysis assesses the extent to which MR expression changes contribute to lifespan extension in PG deletion strains that exhibit prolonged RLS. Panels (A), (B), and (C) correspond to contribution thresholds of 0.3, 0.5, and 0.7, respectively. And top10 contributing MRs for each PG were chosen, representing the minimum contribution score required to classify a PG–MR relationship as functionally relevant. For each threshold, the figure displays the proportion of long-lived PG mutants in which the identified MRs show consistent expression changes associated with RLS extension, thereby providing evidence for regulatory interactions between PGs and MRs. The use of multiple thresholds demonstrates the robustness of the observed PG–MR associations across varying stringency levels.

**Supplementary Figure 5**


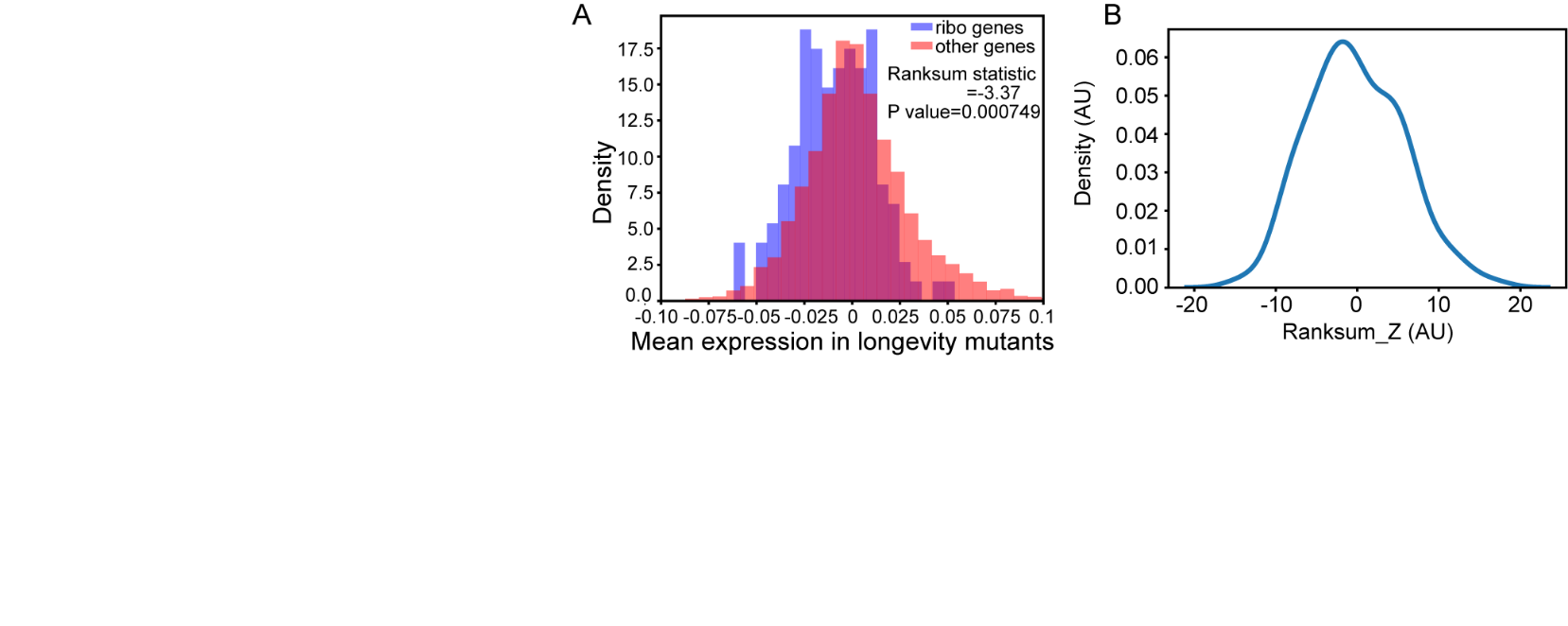


**Supplementary Figure 5. Ribosome gene expression is commonly downregulated in longevity mutants**Expression levels of all genes in the whole genome were ranked in all longevity mutants (see Methods). The expression level of ribosomal genes was significantly lower compared to the control group. Z-score analysis for each mutant shows a normal distribution, indicating that modulation of ribosome pathways is a common feature of longevity.

**Supplementary Figure 6**


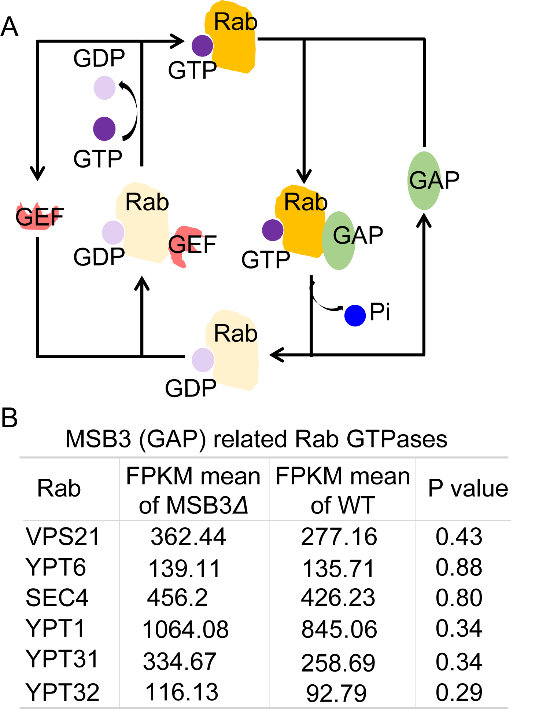


**Supplementary Figure 6. Regulatory relationship and expression of MSB3 and its related GTPases**
(A) Schematic diagram of the regulatory relationship between MSB3 (a GAP) and its associated GTPases. (B) Expression profiles of the enzymes involved in the MSB3-related GTPase regulatory network.
